# Case Report: Endothelial-targeted bridging therapy for a TTP-like phenotype in fulminant iMCD-TAFRO

**DOI:** 10.3389/fimmu.2026.1776382

**Published:** 2026-02-26

**Authors:** Jing Du, Liangliang Wu, Haigang Li, Haibo Gan, Tao Yu, Shuangfeng Xie, Xiangshao Fang

**Affiliations:** 1 Department of Emergency Medicine, Sun Yat-sen Memorial Hospital, Sun Yat-sen University, Guangzhou, China; 2 Department of Pathology, Sun Yat-sen Memorial Hospital, Sun Yat-sen University, Guangzhou, China; 3 Department of Emergency Medicine, Huidong County People’s Hospital, Huizhou, China; 4 Department of Hematology, Sun Yat-sen Memorial Hospital, Sun Yat-sen University, Guangzhou, China

**Keywords:** case report, endotheliopathy, idiopathic multicentric castleman disease, iMCD−TAFRO, interleukin-6 inhibition, therapeutic plasma exchange, thrombotic microangiopathy, TTP−like thrombotic microangiopathy

## Abstract

**Background:**

iMCD-TAFRO (the TAFRO clinical subtype of idiopathic multicentric Castleman disease (iMCD)) is characterized by thrombocytopenia, anasarca, fever, reticulin fibrosis, and organomegaly. Diagnosis is challenging due to its rarity, non-specific early presentation, and overlap with sepsis, lymphoma, and thrombotic microangiopathy (TMA). Endothelial injury is increasingly recognized as a central driver of its severe complications.

**Case presentation:**

A previously healthy 20-year-old woman presented with rapid progression of fever, anasarca, jaundice, and respiratory failure. Laboratory findings revealed severe thrombocytopenia, microangiopathic hemolytic anemia, markedly elevated IL-6, and critically reduced ADAMTS13 activity (5.41%) without an inhibitor by a Bethesda assay, in a sample obtained before the first therapeutic plasma exchange (TPE) and before any fresh frozen plasma (FFP) infusion, suggesting a cytokine-driven thrombotic thrombocytopenic purpura (TTP)-like syndrome. Extensive workup excluded primary infections and malignancies. Diagnosis of iMCD-TAFRO was confirmed by a lymph-node core biopsy showing features consistent with Castleman disease with plasmacytosis.

**Conclusion:**

This case highlights that iMCD-TAFRO can manifest as a fulminant, endotheliopathy-dominated syndrome in young adults, mimicking primary TTP. The severe ADAMTS13 deficiency in this context likely results from consumption due to endothelial activation rather than an immune-mediated process. This distinction is critical for appropriate management.

**Management and Outcome:**

Prior to definitive diagnosis, an endothelium-directed bundle was initiated, including TPE plus adjunctive FFP infusion, corticosteroids, and anisodamine for microcirculatory support; stabilization occurred in the context of multimodal supportive care. Upon diagnostic confirmation, targeted anti-IL-6 therapy (siltuximab) led to significant clinical improvement. Despite a subsequent relapse with pulmonary hypertension, intensified immunosuppression achieved complete remission at one-year follow-up. This case illustrates a pathophysiology-informed bridging bundle to stabilize endothelial and microcirculatory dysfunction while pursuing definitive diagnosis and targeted cytokine blockade in severe iMCD-TAFRO.

## 1 Introduction

iMCD-TAFRO (the TAFRO clinical subtype of idiopathic multicentric Castleman disease (iMCD)), a rare and life-threatening condition, presents a critical diagnostic and therapeutic challenge in acute care settings. Its classic pentad of symptoms—thrombocytopenia, anasarca, fever, reticulin fibrosis, and organomegaly (1–3)—often emerges explosively against a background of non-specific prodromal symptoms. While cohort studies describe a middle-aged, male-predominant demographic with infrequent pulmonary involvement (2), the true incidence is likely underestimated, especially in young adults, owing to low clinician awareness and the syndrome’s masterful mimicry of more common conditions like sepsis, lymphoma, and hemophagocytic lymphohistiocytosis (HLH).

A particularly daunting diagnostic dilemma arises when a suspected iMCD-TAFRO presentation manifests with a thrombotic microangiopathy (TMA) phenotype and severe ADAMTS13 (a disintegrin and metalloproteinase with thrombospondin type 1 motif, member 13) activity deficiency, blurring the line with thrombotic thrombocytopenic purpura (TTP). This distinction is not academic but profoundly therapeutic: while plasma exchange is definitive for immune-mediated TTP, its role in cytokine-driven, secondary TMA is primarily supportive, with disease control hinging on timely IL-6 pathway blockade. The urgency of this decision is compounded by the syndrome’s rapid progression to multi-organ failure, creating a narrow window where diagnostic uncertainty clashes with the imperative for life-supporting intervention.

Emerging evidence positions cytokine storm-mediated endothelial injury as the central pathophysiology (4), a mechanism underpinning the profound capillary leak and thrombotic microangiopathy that define iMCD-TAFRO (2). This insight, however, begs a pivotal clinical question: how can we leverage this knowledge to stabilize patients during the perilous diagnostic interval, before definitive tissue diagnosis and targeted therapy are secured? We propose that an endothelium-targeting, pathophysiology-informed bundle could serve as a hypothesis-generating bridge to support organ function while diagnostic clarification is pursued.

Here, we report the case of a previously healthy 20-year-old woman who presented with a fulminantsuspected iMCD-TAFRO variant, notable for its pronounced pulmonary involvement, cholestatic liverinjury, and a profound TTP-like syndrome. This case exemplifies the diagnostic complexity at the intersection of hyperinflammation and endotheliopathy. We describe a stepwise diagnostic approach centered on image-guided lymph node core biopsy when excision was unfeasible (**Supplementary Figure S1**). Furthermore, we describe an ICU-based multimodal bundle for endothelial and microcirculatory stabilization. This case highlights endotheliopathy in severe iMCD-TAFRO and a hypothesis-generating bridging approach during diagnostic workup and cytokine blockade.

## 2 Case presentation

A previously healthy 20-year-old Chinese woman first noticed left cervical lymphadenopathy 5 weeks prior to admission to our hospital. Initial evaluation at a local hospital revealed complete blood count (CBC) showing white blood cell count (WBC) 8.26×10^9^/L, neutrophils 68.8%, hemoglobin (HGB) 156 g/L, platelet count (PLT) 266×10^9^/L, and elevated C-reactive protein (14.54 mg/L; reference range, 0 to 5). Cervical ultrasound demonstrated multiple hypoechoic lymph nodes measuring 11×7mm to 14×7mm with homogeneous internal echoes and minimal vascular flow. The lymphadenopathy temporarily improved with traditional Chinese medicine treatment, but the patient subsequently developed posterior back pain, fever (maximum 38 °C), abdominal distension, and poor appetite.

**Figure 1:**
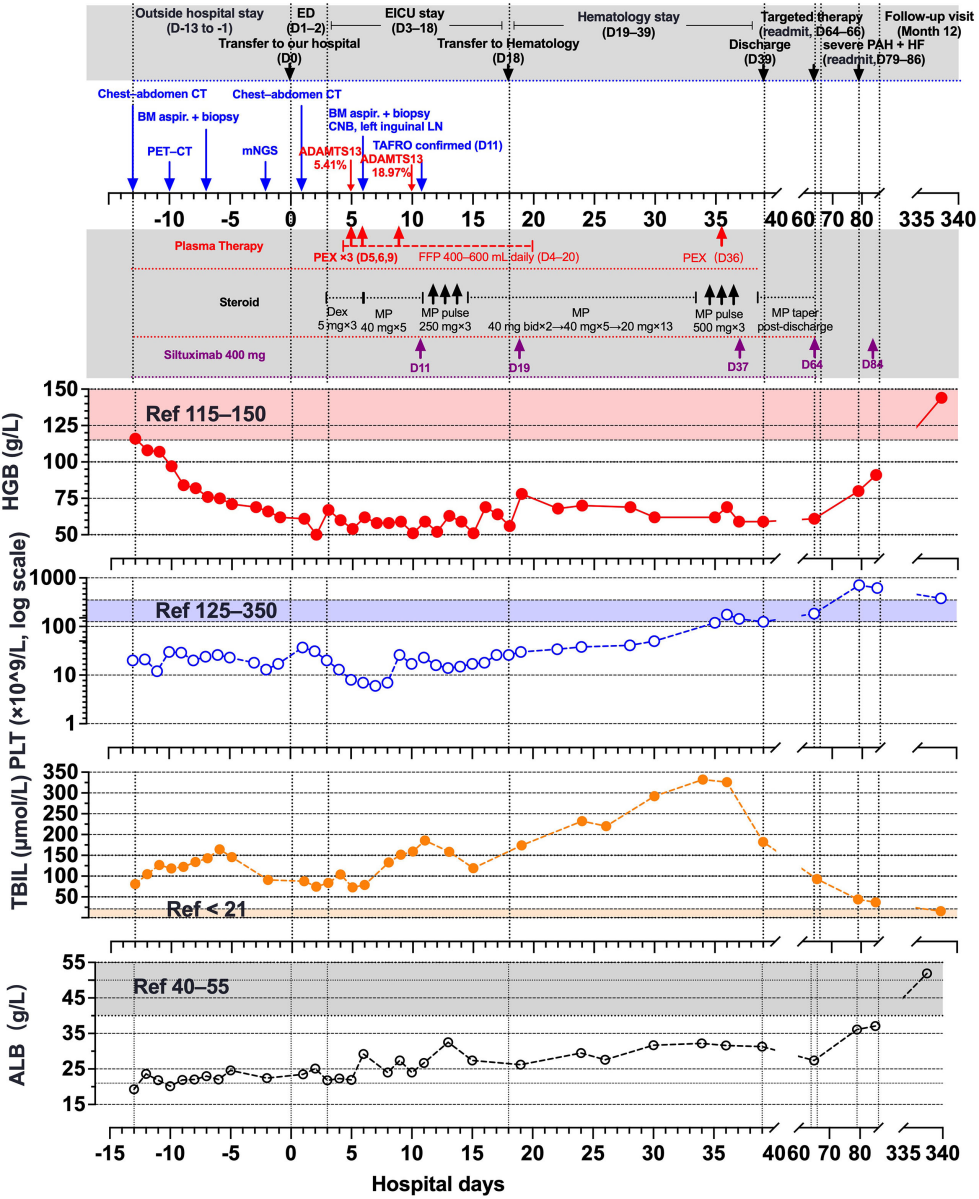
Clinical timeline, laboratory trajectories, and therapies across the index admission and follow-up. The timeline is anchored to admission at our center (D 0). Negative values denote pre-admission days; positive values indicate days after admission. Platelets are plotted on a logarithmic scale. HGB and platelet count are highlighted because they are commonly used on-treatment response markers for siltuximab in iMCD, and early improvement is typically observed within the first several doses in responders (3, 21). TBIL is included because cholestatic hyperbilirubinemia was a major organ dysfunction feature in this case. ALB is included because hypoalbuminemia is a minor diagnostic criterion for iMCD and was serially monitored in parallel with TBIL during follow-up. ALB, albumin; ED, Emergency Department; EICU, Emergency Intensive Care Unit; PAH, pulmonary arterial hypertension; HF, heart failure; mNGS, metagenomic next-generation sequencing; BM, bone marrow; CNB, core-needle biopsy; LN, lymph node; Dex, dexamethasone; MP, methylprednisolone; PEX, plasma exchange; FFP, fresh frozen plasma.

Two weeks before admission to our hospital, the patient presented to a regional tertiary hospital emergency department (ED) with progressive dyspnea, jaundice, and bilateral leg edema, and remained under ED observation for 13 days. The pre-admission course and the index hospitalization are summarized in the timeline (**Figure 1**). Initial labs showed leukocytosis with neutrophilia (WBC 14.59×10^9^/L; 84.4% neutrophils), thrombocytopenia (PLT 20×10^9^/L), mild anemia (HGB 116 g/L), markedly elevated inflammatory markers (hsCRP 180.4 mg/L, procalcitonin 9.91 ng/mL, IL−6 133 pg/mL), hypoalbuminemia (19.26 g/L), and hyperbilirubinemia (total/direct 80.9/57.2 μmol/L). Chest CT revealed bilateral multifocal ground-glass opacities with small consolidations, crazy-paving, and peribronchovascular thickening; mildly enlarged, partially confluent mediastinal lymph nodes; and small pericardial and bilateral pleural effusions. Over 13 days, the patient developed progressive bicytopenia (PLT nadir 12×10^9^/L, HGB 62 g/L), persistent leukocytosis (WBC 23.65×10^9^/L), coagulopathy (D-dimer >20,000 ng/mL), and worsening organ dysfunction (bilirubin 164.2 μmol/L, NT-proBNP 7091 pg/mL).

The patient’s rapid deterioration with severe thrombocytopenia, anemia, marked systemic inflammation, and multi-organ involvement necessitated urgent differential diagnosis including severe infection/sepsis, hyperinflammatory syndromes (e.g., HLH-spectrum disease), and hematologic malignancy.

## 3 Diagnostic reasoning and differential diagnosis

### 3.1 Excluding infection: conventional microbiology vs blood mNGS

Extensive infectious disease evaluation showed negative results on repeated blood cultures, fungal β-D-glucan assay, respiratory pathogen nucleic acid testing covering common bacteria (*Streptococcus pneumoniae, Staphylococcus aureus, Klebsiella pneumoniae, Pseudomonas aeruginosa*), mycobacterial studies for both *M. tuberculosis* and *non-tuberculous mycobacteria*, and the T-SPOT assay; however, metagenomic next-generation sequencing (mNGS) of peripheral blood detected *Human betaherpesvirus 5* (CMV), *Human gammaherpesvirus 4* (EBV), and *Ureaplasma urealyticum* with high identification confidence (99%) and varying sequence reads. The discordance between negative conventional microbiology and positive blood mNGS most likely reflects low-level viremia/reactivation (EBV, CMV) or colonization (Ureaplasma) rather than pathogen-driven sepsis. Overall, the findings favor a non-infectious, cytokine-mediated inflammatory process—with possible viral reactivation as a bystander/contributor—rather than an infection-driven inflammatory syndrome.

### 3.2 Considering malignancy: imaging and laboratory features arguing against aggressive lymphoma

Lymphoma was initially suspected given progressive lymphadenopathy, fever, and bicytopenia.However, the ^18^F-FDG PET/CT performed pre-transfer at a regional tertiary hospital EDdemonstrated disseminated lymphadenopathy (involving the cervical/supraclavicular, mediastinal, hilar, axillary, retroperitoneal, mesenteric, iliac, and inguinal stations; measuring 0.9–2.2 cm) with mild-to-moderate heterogeneous uptake (SUVmax 1.9–5.7), bilateral pulmonary lesions with increased glucose uptake, and hepatosplenomegaly (spleen measuring 7.9 cm in vertical length) with decreased hepatic metabolism suggestive of impaired liver function, without a pattern strongly supportive of an aggressive lymphomatous process (Nodal measurements are summarized in **Supplementary Table S1a**; representative PET/CT images are shown in **Supplementary Figure S2**.). Serial LDH values remained within the reference range—features that, while not excluding indolent or early-stage lymphoma, argue against a high-turnover, aggressive lymphomatous process (5–7). Together with a non-diagnostic bone marrow evaluation, these findings made high-grade lymphoma less likely and favored an inflammatory rather than neoplastic etiology.

### 3.3 Bone marrow findings: reactive myelofibrosis vs clonal myeloid neoplasm

Bone marrow biopsy revealed hypercellular marrow with morphologically normal megakaryocytes. Immunohistochemical analysis demonstrated negative staining for CD117, CD20, CD3, CD34, and TdT, with negative EBERs by *in situ* hybridization, making acute leukemia and lymphoma less likely, while MPO positivity confirmed increased granulocytic lineage and CD42b positivity validated megakaryocyte proliferation. Reticulin silver stain demonstrated severe reticulin fibrosis (WHO MF Grade 3); Masson trichrome revealed severe collagen deposition (grade 3) with mild osteosclerosis (grade 1); Berlin blue stain was negative. The pathologist raised the possibility of myelodysplastic neoplasm (MDS) or myelodysplastic/myeloproliferative neoplasm (MDS/MPN) pending clinical correlation; however, the combination of MF-3 fibrosis with otherwise preserved trilineage hematopoiesis is more consistent with cytokine-driven reactive myelofibrosis than a clonal MPN or MDS/MPN. Therefore, a clonal myeloid neoplasm should not be presumed without further workup, including cytogenetics, a myeloid NGS panel, and expert morphologic (± flow cytometric) re-evaluation to assess clonality and dysplasia.

### 3.4 Tertiary referral and ICU presentation: hyperinflammation with capillary leak

Due to rapidly worsening anasarca, marked abdominal distension, fever, tachypnea, and dyspnea with an inconclusive diagnosis, the patient was transferred to our tertiary university-affiliated hospital for advanced evaluation and care. On arrival (day 1), she underwent comprehensive assessment in the Emergency Department for 2 days and was admitted to the Emergency ICU (EICU) on day 3.

**Figure 2:**
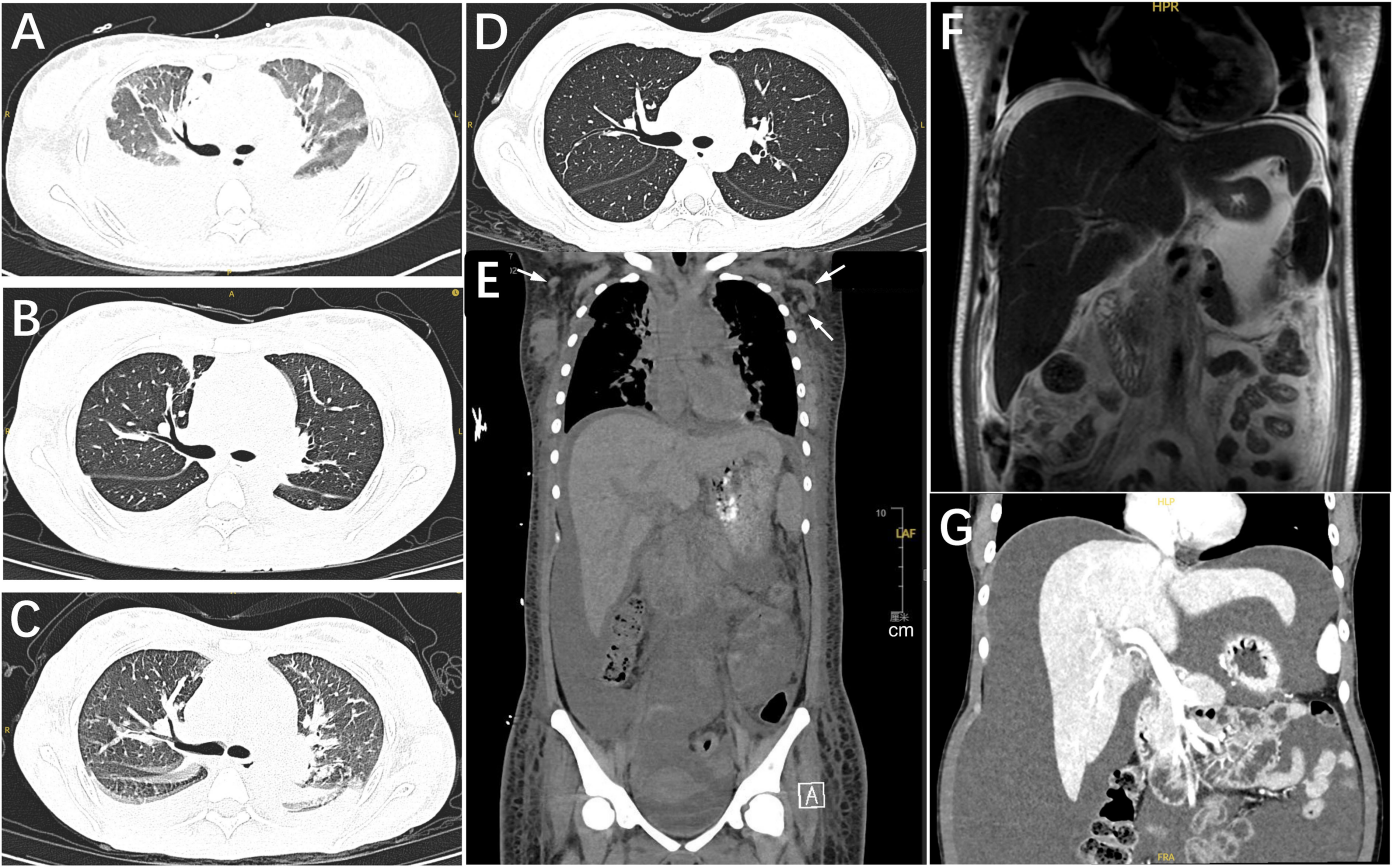
Serial thoracoabdominal imaging showing dynamic anasarca, pleuro−pulmonary involvement,and serous effusions. Cross−sectional studies are aligned to days from index admission (D 0).**(A)** Chest CT (D 1): Multifocal inflammatory pulmonary opacities; moderate bilateral pleural effusions including interlobar fissural fluid. **(B)** Chest CT (D 65): Decreased pulmonary opacities and pleural effusions versus D 1. **(C)** Chest CT (D 79): Radiographic pulmonary edema; scattered inflammatory opacities and pleural effusions slightly increased versus D 65, during clinical PAH/HF. **(D)** Chest CT (D 126): Near−complete resolution of pulmonary opacities and pleural effusions. **(E)** Abdominopelvic CT (D 1): Moderate ascites; diffuse thoracoabdominal wall edema; axillary lymph nodes (arrows) without a dominant mass (scale bar shown). Axillary nodal stations and exact short-axis diameters documenting multicentric lymphadenopathy are provided in **Supplementary Table S1a**. **(F)** Abdominal MRI (D 36): Coronal T2−weighted image shows small−volume ascites and diffuse subcutaneous edema. **(G)** Abdominal CT (D 137): Large−volume ascites, increased versus prior imaging. CT, computed tomography; MRI, magnetic resonance imaging; T2WI, T2−weighted image; D, day relative to admission; PAH, pulmonary arterial hypertension; HF, heart failure; ED, Emergency Department.

Initial testing confirmed critical illness. CBC showed leukocytosis (WBC 19.86×10^9^/L with 90.1% neutrophils), severe anemia (HGB 60 g/L), and severe thrombocytopenia (PLT 13×10^9^/L). Liver injury with hypoproteinemia (albumin 22.3 g/L), conjugated hyperbilirubinemia (total/direct: 103.9/58.6 μmol/L), elevated ALP (457 U/L), and GGT (111 U/L) was present. Renal function showed mild impairment (creatinine 109 μmol/L; estimated glomerular filtration rate [eGFR] 65 mL/min/1.73 m^2^, CKD-EPI 2021 equation for a 20-year-old female). Inflammatory markers were high (procalcitonin, ferritin, sIL-2R, IL-6, IL-10, TNF-α; **Table 1**). CT revealed multifocal bilateral pulmonary inflammatory opacities; generalized lymphadenopathy (axillary, mediastinal, hilar, retroperitoneal); serous effusions (pericardial, pleural, peritoneal, pelvic); and diffuse subcutaneous edema of the chest and abdominal wall (**Figures 2A, E**). On EICU admission (day 3), vitals showed fever 39°C, tachypnea 44 breaths/min, and tachycardia 140 beats/min. Examination noted jaundice; diffuse petechiae and ecchymoses; marked abdominal distension; severe pitting edema with cool extremities; and multiple mobile lymph nodes in the left cervical and bilateral inguinal regions.

**Table 1:** Inflammatory markers across the clinical course.

Parameter	Day 4	Day 14	Day 35	Day 66	Day 80	Day 137	Day 340	Reference range
sIL-2R	793	426	299	460	284	129	170.0	223 to 710
IL-6	11.9	8.8	14.0	15.6	10.4	4.8	6.3	0.0 to 5.9
IL-10	37.7	9.2	<5.0	23.4	<5.0	<5.0	<5.0	0.0 to 9.1
TNF-α	37.1	22.6	18.3	32.0	28.3	13.4	11.4	0.0-8.1
Procalcitonin	8.65	5.24	—	—	1.46	—	—	0-0.10
ferritin	500	1122	1646	—	1449	—	—	6-159

Reference ranges are shown in the rightmost column. “—” indicates not obtained. Units: sIL−2R (U/mL), IL−6/IL−10/TNF−α (pg/mL), procalcitonin (ng/mL), ferritin (ng/mL).

### 3.5 TTP-like microangiopathy: ADAMTS13 results and etiologic re-classification

Given fever, severe thrombocytopenia (nadir 6×10^9^/L), and microangiopathic hemolytic anemia (HGB nadir 50 g/L), a diagnosis of TTP or a TTP-like syndrome was considered, prompting ADAMTS13 activity and inhibitor testing (8). On day 5, ADAMTS13 activity was markedly reduced (5.41%; reference range ≥70%; TTP threshold <10%) as measured by a fluorescence resonance energy transfer assay, and the inhibitor was negative by a Bethesda assay (0.00 BU; reference <0.6), making classic immune-mediated TTP less likely. The blood sample for ADAMTS13 testing was collected before the first therapeutic plasma exchange (TPE) and any fresh frozen plasma (FFP) exposure, minimizing treatment-related effects on activity. Together with the absence of typical TTP-dominant features (notably focal neurologic deficits and disproportionate acute kidney injury) and a clinical course dominated by hyperinflammation and endothelial activation, these findings supported cytokine-driven endothelial injury with secondary thrombotic microangiopathy (TMA) and capillary-leak physiology; accordingly, management prioritized control of hyperinflammation/endothelial injury with TMA-directed supportive care.

### 3.6 Parallel diagnostic workup and diagnostic confirmation: lymph-node pathology and iMCD-TAFRO criteria

On day 6, bone marrow aspirate was obtained for an MDS-focused myeloid gene panel, which returned negative one week later. In parallel, an ultrasound-guided core needle biopsy (CNB) of a left inguinal lymph node was performed, with the specimen was allocated to (1) flow-cytometric immunophenotyping for CLL/lymphoma, showing no abnormal lymphoid population; (2) metagenomic next-generation sequencing for broad pathogen detection (bacteria, fungi, DNA viruses, and parasites), which was negative; and (3) histopathology, which was pending because of prolonged processing.

**Figure 3:**
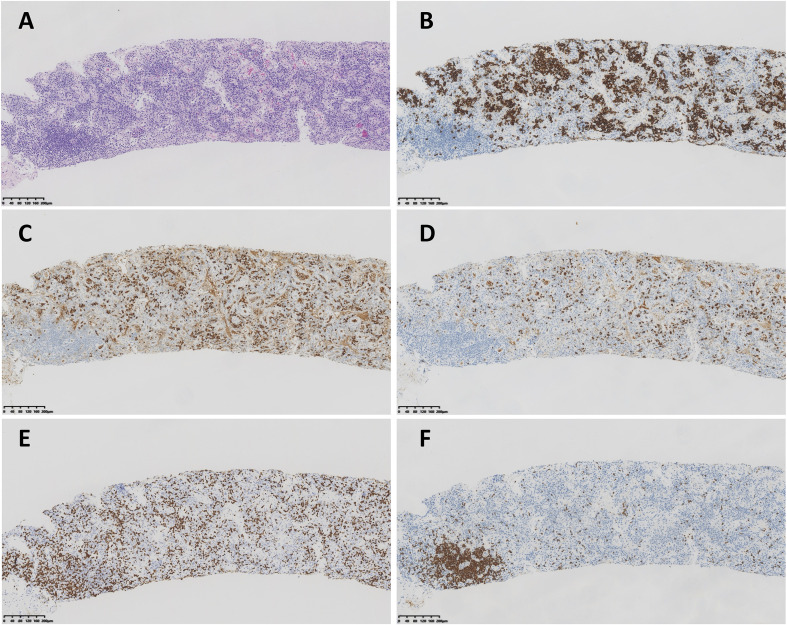
Lymph−node core−needle biopsy consistent with multicentric Castleman disease. **(A)** Hematoxylin–eosin stain shows regressed/partially atrophic germinal centers with vascular proliferation and dilated sinuses, with marked interfollicular plasmacytosis. **(B)** CD138 highlight abundant plasma cells. **(C, D)** κ and λ light−chain immunostains demonstrate polytypic (polyclonal) expression without restriction. **(E)** CD3 showed T cells. **(F)** CD20 showed aggregation of B cells suggested the regressed follicle.

The diagnostic breakthrough came on day 10, when a lymph−node core biopsy revealed findings consistent with Castleman disease (**Figures 3A–F**): regressed germinal centers with prominent vascular proliferation and dilated lymphaticsinuses; marked interfollicular plasmacytosis with CD38/CD138–positive plasma cells showingpolyclonal κ and λ light−chain expression without restriction; preserved follicular dendritic cell networks (CD21−positive); relatively sparse T− and some B−cell populations; a proliferation index (Ki−67) of approximately 30%; and EBER *in situ* hybridization negative. Integrated with the clinical syndrome, these histopathologic and immunophenotypic findings suggested multicentric Castleman disease. No positive result was found in Qualitative real-time PCR for HHV-8 DNA in plasma. Subsequently, hematologic assessment confirmed that all iMCD−TAFRO criteria were fulfilled: Thrombocytopenia (T) with nadir 6×10^9^/L; Anasarca (A) with generalized edema and multiple effusions; persistent Fever (F) up to 39 °C; Reticulin fibrosis (R) in pathology of bone marrow biopsy; Organomegaly (O) with hepatomegaly and lymphadenopathy (see **Supplementary Tables S1**, **S2**). Key mimics were excluded (see **Supplementary Table S3**). The final diagnosis came out: idiopathic multicentric Castleman disease (iMCD), HHV−8–negative, TAFRO clinical subtype, severe, fulfilling the 2017 iMCD diagnostic criteria and the 2021 validated international definition of iMCD-TAFRO (2, 3, 9).

## 4 Endothelium targeted bundle therapy

Given the markedly reduced ADAMTS13 activity (<10%), caplacizumab was considered per ISTH guidance for suspected iTTP (10). It was not administered because the overall clinicopathologic picture favored a cytokine-driven secondary TMA/TTP-like syndrome with likely consumptive ADAMTS13 reduction, and caplacizumab is not currently available in mainland China.

Upon admission to the EICU (day 3), a multimodal, pathophysiology-informed bridging bundle was initiated to stabilize organ function amid endotheliopathy and microcirculatory failure: (1) ADAMTS13 supplementation via TPE (3 sessions of 2000 mL on days 5, 6, and 9) followed by daily FFP infusion (400–600 mL)for 14 days (excluding exchange days); (2) anti−inflammatory therapy with dexamethasone 5 mg daily ×3 (days 4–6) then methylprednisolone 40 mg daily ×5 (days 7–11); (3) microcirculatory support with continuous anisodamine (an M-cholinergic receptor antagonist) infusion (10–30 mg/h, days 8–10) plus intermittent furosemide (10–20 mg/h) with monitoring of peripheral temperature and urine output. In our setting, anisodamine is routinely available; in this case, it was administered off-label with institutional approval and informed consent (not compassionate use). Concurrently, node core specimens were sent for pathology, immunophenotyping and mNGS as described.

Anisodamine was used as an adjunct for microcirculatory support with continuous ECG and hemodynamic monitoring. Evidence from multicenter RCTs in septic shock remains mixed (11, 12). Therefore, we report only a temporal association in this case. During infusion, anticholinergic effects occurred (bilateral mydriasis, pupils 2.5 mm pre-treatment to 4–8 mm) with a modest increase in sinus tachycardia (~10–15 beats/min), managed with metoprolol (12.5 mg twice daily). Bowel sounds were not further suppressed, and no other serious adverse events were observed.

After eight days of the bundle (days 3–10), physiologic changes were observed: ADAMTS13 activity increased from 5.41% (day 5) to 18.97% (day 10); toe temperature rose from 28.0 °C pre-treatment to 36.5–37.5 °C during anisodamine infusion (within the multimodal bundle); mean daily urine output increased from 2,810 mL (day 7) to 3,816 mL/day during days 8–10, with an average daily negative fluid balance of −360 mL/day. These changes prompted a mechanistic interpretation of the bundle’s potential effects on endotheliopathy and microcirculatory failure.

Faced with cytokine storm–associated endotheliopathy with TTP-like features and capillary leak, we implemented a pathophysiology-driven supportive bundle combining ADAMTS13 replacement (TPE/FFP), anti-inflammation (steroids), and adjunctive microcirculatory support (anisodamine ± diuresis). Within this bundle, TPE/FFP and diuresis were used primarily as supportive stabilization measures, corticosteroids as anti-inflammatory therapy that may secondarily mitigate endothelial activation, and anisodamine as an exploratory adjunct for microcirculatory support. Given the mixed randomized evidence for anisodamine in septic shock (11, 12), the observed rapid peripheral warming provides only a physiologic signal plausibly consistent with improved microvascular perfusion (13). Although concurrent improvements in toe temperature, urine output, and ADAMTS13 activity support partial reversal of endothelial dysfunction, they do not establish causality; thus, this strategy warrants systematic evaluation rather than immediate generalization.

## 5 Cytokine-targeted immunotherapy

After diagnostic confirmation, cytokine-targeted immunotherapy was initiated with siltuximab (400 mg on day 11, re-dosed on day 19, and maintained on day 37). This approach aligned with guideline-recommended anti–IL-6 therapy (standard siltuximab 11 mg/kg IV q3w) (3), with early accelerated weekly dosing considered for critically ill severe iMCD, and subsequent dosing intervals individualized according to clinical response and follow-up feasibility rather than fixed every 3 weeks. High-dose methylprednisolone was administered as pulse therapy (250 mg daily for 3 days, days 12–14), followed by stepwise tapering; a second pulse (500 mg daily for 3 days, days 35–37) was given for disease control with subsequent taper. During hematology care, a short 8-day course of cyclosporine A was added as adjunct immunosuppression.

By day 18, the patient stabilized sufficiently for transfer to hematology, with resolution of fever and dyspnea, improved generalized edema (residual moderate lower-extremity pitting), and restored peripheral perfusion. Platelets improved to 30×10^9^/L, while anemia (hemoglobin 78 g/L), hypoalbuminemia (26.2 g/L), and cholestatic-pattern liver injury (total bilirubin 173.8 μmol/L, GGT 198 U/L) persisted.

At discharge on day 39, systemic symptoms had resolved and edema markedly regressed. Platelets recovered to 125×10^9^/L; anemia (hemoglobin 59 g/L), hypoalbuminemia (31.3 g/L), and cholestasis (total bilirubin 182.1 μmol/L) remained, with preserved hepatocellular enzymes (ALT 17 U/L) and normal renal function (creatinine 45 μmol/L, eGFR 137 mL/min/1.73 m²).

Overall, management appropriately transitioned from empiric endothelial stabilization during diagnostic uncertainty to guideline-consistent cytokine suppression with anti–IL-6 therapy plus corticosteroids for severe iMCD-TAFRO (14), with clinical and hematologic improvement supporting the working diagnosis and highlighting the value of ICU “bridge” care until definitive therapy was feasible.

## 6 Long-term follow-up

Post−discharge, targeted therapy continued with a fourth siltuximab dose (400 mg) on day 64. On day 79, the patient developed severe dyspnea with orthopnea and could not tolerate the supine position, requiring a semi−upright posture. Echocardiography revealed marked elevation in pulmonary artery systolic pressure (PASP 74 mmHg) with reduced ejection fraction (EF 44%), accompanied by leukocytosis, anemia, and marked thrombocytosis (WBC 20.18×10^9^/L, HGB: 80 g/L, PLT 708×10^9^/L). Treatment intensification included cyclophosphamide (400mg IV, day 79) and a fifth siltuximab dose (400 mg, day 84; five doses in total), followed by maintenance therapy with ruxolitinib (5 mg twice daily) and weekly cyclophosphamide (400mg oral), achieving pulmonary pressure normalization by day 126. Brief rehospitalization (days 136-141) was required for paracentesis due to persistent ascites. Serial cross−sectional imaging mirrored the clinical course, demonstrating decreasing pulmonary infiltrates and pleural effusions by day 126 and recurrent large−volume ascites by day 137 (**Figures 2B–D, F, G**).

At one-year follow-up, complete clinical remission was achieved with normal daily functioning, normalized hematological parameters (WBC 6.7×10^9^/L, HGB144 g/L, PLT 380×10^9^/L), restored organ function (albumin 51.9 g/L, total bilirubin 15.6 μmol/L, creatinine 66 μmol/L, eGFR 118 mL/min/1.73 m^2^), complete resolution of ascites, and inflammatory marker normalization (sIL-2R, IL-10) with minimal residual IL-6 and TNF-α elevation (**Table 1**).

## 7 Discussion

This case elucidates a severe iMCD-TAFRO phenotype dominated by cytokine-mediated endotheliopathy, which manifested as fulminant capillary leak and a secondary TMA/TTP-like syndrome. Beyond expanding the clinical spectrum in young adults, it provides a critical framework for managing similar crises, emphasizing the pivotal distinction between primary immune-mediated TTP and cytokine-driven secondary TMA, and illustrating the potential role of a pathophysiology-directed, endothelium-targeted bridging strategy.

The diagnostic challenge resided in the substantial syndrome overlap. While distinctions from myelodysplastic syndromes and lymphoma were necessary, the most consequential dilemma was the profound ADAMTS13 activity deficiency (5.41%) without an inhibitor. This profile, coupled with a dominant hyperinflammatory state, was more consistent with a secondary, inflammation-associated endotheliopathy with consumptive ADAMTS13 reduction than with primary immune TTP, a distinction with immediate therapeutic implications (8, 15). Notably, severe ADAMTS13 reduction may also occur in secondary thrombotic microangiopathy (e.g., sepsis/cytokine storm) via endothelial activation and consumption even when an inhibitor is undetectable (8, 15). Critically, the definitive diagnosis of iMCD-TAFRO was established by integrating the lymph-node histopathology with the characteristic clinical constellation (1, 2), underscoring the necessity of tissue confirmation amidst complex presentations. Consistent with the 2019 updated TAFRO criteria and ongoing taxonomy debate, we use “iMCD-TAFRO” only after iMCD-consistent lymph-node histology is confirmed (16, 17).

This pathophysiological insight directly informed our therapeutic strategy. In immune TTP, plasma exchange is definitive therapy (10); here, we repositioned it as a physiology−informed, supportive−care bridging modality, potentially supplementing ADAMTS13 and mitigating endothelial injury while awaiting diagnostic confirmation. This physiology−informed, hypothesis−generating rationale was based on the possibility of consumptive ADAMTS13 reduction in severe inflammation/secondary TMA (8, 15). The staged approach—prioritizing endothelial and microcirculatory support during the diagnostic window, then rapidly escalating to IL-6 blockade—was crucial. The patient’s rapid response to siltuximab aligns with robust evidence establishing anti-IL-6 therapy as the cornerstone for iMCD-TAFRO (14, 18).

Adjunctive anisodamine was incorporated to address microcirculatory dysfunction. In this patient, its use coincided with improved peripheral perfusion and diuresis; however, given concurrent interventions and mixed RCT results in septic shock, these findings should be interpreted as temporal association rather than evidence of efficacy (11, 12).

The subsequent development of severe pulmonary hypertension underscores the relapsing–remitting nature of TAFRO, necessitating vigilant monitoring and adaptive immunotherapy. Given that pulmonary vascular and parenchymal involvement have been reported in severe TAFRO, this relapse helps place the patient’s lung findings within the iMCD TAFRO spectrum. Although uncommon, pulmonary involvement in severe TAFRO may include acute interstitial lung injury/rapidly progressive ground glass opacities and diffuse alveolar damage, attributed to IL 6 overproduction–associated cytokine storm (19). Furthermore, IL−6/VEGF–driven endothelial dysfunction and vascular hyperpermeability (“endotheliopathy”) have been proposed as central mechanisms in TAFRO (20), providing a plausible link between systemic relapse activity and the reversible pulmonary hypertension observed during follow−up.

From a practical standpoint, this case yields three key transferable insights: (1) Maintain a high index of suspicion for iMCD-TAFRO in hyperinflammatory patients with anasarca, cytopenias, and TMA, even with subtle lymphadenopathy (1–3), and prioritize early tissue confirmation when feasible. (2) Urgently differentiate the underlying mechanism of TMA/TTP-like syndromes using ADAMTS13 activity/inhibitor testing (ideally sampled before plasma therapy) and inflammatory context, as this distinction critically determines whether plasma exchange serves as definitive therapy (for immune TTP) or as a bridging supportive measure (in cytokine-driven endotheliopathy) (8, 10). (3) Implement integrated workflows between hematology and intensive care units to synchronize tissue diagnosis and inflammatory profiling with concurrent physiological stabilization, thereby minimizing critical treatment delays, and enabling timely transition to IL−6 pathway blockade once iMCD−TAFRO is established.

Limitations inherent to a single-case report include the inability to establish causality for adjunctive treatments and incomplete biomarker serialization. In addition, although excisional lymph-node biopsy is preferred in iMCD, it was not feasible given the patient’s critical condition with marked edema and small/deep lymph nodes; therefore, ultrasound-guided core needle biopsy (CNB) was chosen as the safest practical option. Despite CNB’s limitation in assessing nodal architecture, the core biopsy demonstrated iMCD-consistent features (**Figures 3A–F**) and, when integrated with the characteristic clinical constellation and exclusion workup, supported the diagnosis of iMCD-TAFRO in this patient.

In conclusion, this report illustrates a structured clinical approach for fulminant iMCD-TAFRO. By recognizing cytokine-driven endotheliopathy as a key mechanism, using an endothelium- and microcirculation-targeted supportive bridging strategy during the diagnostic window, and ensuring timely transition to definitive IL-6 blockade, clinicians may better navigate this high-risk phase. Although causality cannot be established from a single case, this multidisciplinary, pathophysiology directed framework provides a pragmatic reference for managing similar crises and warrants further evaluation.

## 8 Patient perspective

Patient perspective could not be obtained due to critical illness during the ICU stay; no subsequent narrative was available at the time of submission.

## Data Availability

The original contributions presented in the study are included in the article/**Supplementary Material**. Further inquiries can be directed to the corresponding author.

## Supplementary material

Supplementary Figure 1 Diagnostic and initial management algorithm for hyperinflammatory TMA/TTP-like presentations with parallel evaluation for iTTP and iMCD/iMCD-TAFRO. Stepwise workflow for patients presenting with TMA/TTP-like features and hyperinflammation. Management is divided into pre-ADAMTS13 (while results are pending) and post-ADAMTS13 (after results return) phases. The pathway integrates early iTTP-directed therapy when clinical suspicion is high, parallel exclusion of secondary TMA causes, and expedited evaluation for iMCD/iMCD-TAFRO (including lymph-node biopsy when feasible) to guide subsequent iTTP- or iMCD-directed treatment escalation. Abbreviations: as defined in the main text and Figure legends.

Supplementary Figure 2 Representative chest and abdominopelvic PET/CT images captured from a smartphone medical imaging application.

Supplementary Table S1 Cross-walk to 2017 International iMCD diagnostic criteria. *Adapted from Fajgenbaum et al. Blood. 2017* (3).

Supplementary Table S1 Pre-transfer PET/CT report–derived lymph node stations and short-axis diameters supporting multicentric lymphadenopathy. Footnote: Short-axis was defined as the smaller diameter on CT. Per the 2017 international iMCD diagnostic criteria, multicentric lymphadenopathy requires enlarged lymph nodes (≥1.0 cm short-axis) in ≥2 nodal stations; this requirement was met (e.g., right cervical and mediastinal/para−aortic nodes).

Supplementary Table S2 Cross-walk to 2021 validated international definition of iMCD-TAFRO. *Adapted from Nishimura et al., Am J Hematol 2021* (2).

Supplementary Table S3 Focused exclusion checklist for iMCD differential diagnoses and mimics (malignant/autoimmune/infectious). *Adapted from Fajgenbaum et al., Blood. 2017* (3), *and the Castleman Disease Collaborative Network (CDCN) consensus diagnostic criteria checklist for iMCD (KnowiMCD)* (9). Abbreviations: HL, Hodgkin lymphoma; NHL, non-Hodgkin lymphoma; CLL, chronic lymphocytic leukemia; LPD, lymphoproliferative disorder; LN, lymph node; FC, flow cytometry; EBV, Epstein–Barr virus; EBER-ISH, EBV-encoded RNA *in situ* hybridization; BM, bone marrow; ISH, *in situ* hybridization; PCN, plasma cell neoplasm; MM, multiple myeloma; POEMS, polyneuropathy–organomegaly–endocrinopathy–monoclonal protein–skin changes; IFE, immunofixation electrophoresis; PCs, plasma cells; MDS, myelodysplastic syndrome; MPN, myeloproliferative neoplasm; NGS, next-generation sequencing; HHV-8, human herpesvirus 8; MCD, multicentric Castleman disease; qPCR, quantitative polymerase chain reaction; NAAT, nucleic acid amplification test; mNGS, metagenomic next-generation sequencing; CMV, cytomegalovirus; SLE, systemic lupus erythematosus; CTD, connective tissue disease; ANA, antinuclear antibody; ENA, extractable nuclear antigen; anti-dsDNA, anti-double-stranded DNA; ANCA, anti-neutrophil cytoplasmic antibody; MPO, myeloperoxidase; PR3, proteinase 3.
