# Supplementary material for: Case Report: Endothelial-targeted bridging therapy for a TTP-like phenotype in fulminant iMCD-TAFRO
Source: Front Immunol. 2026 Feb 26;17:1776382. doi: 10.3389/fimmu.2026.1776382 (PMC12979455; doi:10.3389/fimmu.2026.1776382)
Supplement: Supplementary Figure 1 — Diagnostic and initial management algorithm for hyperinflammatory TMA/TTP-like presentations with parallel evaluation for iTTP and iMCD/iMCD-TAFRO. Stepwise workflow for patients presenting with TMA/TTP-like features and hyperinflammation. Management is divided into pre-ADAMTS13 (while results are pending) and post-ADAMTS13 (after results return) phases. The pathway integrates early iTTP-directed therapy when clinical suspicion is high, parallel exclusion of secondary TMA causes, and expedited evaluation for iMCD/iMCD-TAFRO (including lymph-node biopsy when feasible) to guide subsequent iTTP- or iMCD-directed treatment escalation. Abbreviations: as defined in the main text and Figure legends. [file SupplementaryFile1.zip › Table S3.docx]

**Supplementary Table S3. Focused exclusion checklist for iMCD differential diagnoses and mimics (malignant / autoimmune / infectious)**

| **Category** | **Key mimics to exclude** | **Tests performed / key negative (or non-explanatory) findings in this case** | **Conclusion** |
| --- | --- | --- | --- |
| **Malignant** | Lymphoma (Hodgkin lymphoma [HL] / non-Hodgkin lymphoma [NHL]), chronic lymphocytic leukemia (CLL)/other clonal lymphoproliferative disorder (LPD) | Lymph node (LN) core biopsy flow cytometry (FC; CLL/lymphoma panel): no abnormal lymphoid population | Not supported |
|  | EBV-associated LPD | EBV-encoded RNA in situ hybridization (EBER-ISH) negative (LN; also negative in bone marrow [BM] by ISH) | Excluded |
|  | Plasma cell neoplasm (PCN)/multiple myeloma (MM)/plasmacytoma (incl. POEMS driver) | Serum immunofixation electrophoresis (IFE) negative (no M-protein); LN plasma cells (PCs) polyclonal κ/λ | Excluded |
|  | Myelodysplastic syndrome (MDS)/MDS–myeloproliferative neoplasm (MDS-MPN) | Myeloid next-generation sequencing (NGS; MDS-focused panel) negative | Not supported |
| **Infectious** | HHV-8–associated multicentric Castleman disease (HHV-8–MCD) | Plasma HHV-8 DNA quantitative PCR (qPCR) negative | Excluded |
|  | Occult infection mimicking cytokine storm/sepsis | Extensive conventional microbiology negative (incl. repeated blood cultures, fungal β-D-glucan, respiratory pathogen NAAT, mycobacterial studies, and T‑SPOT). Peripheral-blood metagenomic NGS (mNGS) detected CMV/EBV/*Ureaplasma urealyticum* with high confidence, but was discordant with negative routine testing and overall clinical context, favoring low-level viral reactivation/colonization rather than pathogen-driven sepsis | Not supported |
| **Autoimmune / rheumatologic** | Systemic lupus erythematosus (SLE)/connective tissue disease (CTD) | ANA/ENA/anti-dsDNA negative; clinical picture did not meet criteria for systemic autoimmune disease | Excluded |
|  | Vasculitis | Anti-neutrophil cytoplasmic antibody (ANCA; MPO/PR3) negative; no clinical evidence of vasculitis | Excluded |

*Adapted from Fajgenbaum et al., Blood. 2017[3], and the Castleman Disease Collaborative Network (CDCN) consensus diagnostic criteria checklist for iMCD (KnowiMCD)[9].*

**Abbreviations:** HL, Hodgkin lymphoma; NHL, non-Hodgkin lymphoma; CLL, chronic lymphocytic leukemia; LPD, lymphoproliferative disorder; LN, lymph node; FC, flow cytometry; EBV, Epstein–Barr virus; EBER-ISH, EBV-encoded RNA in situ hybridization; BM, bone marrow; ISH, in situ hybridization; PCN, plasma cell neoplasm; MM, multiple myeloma; POEMS, polyneuropathy–organomegaly–endocrinopathy–monoclonal protein–skin changes; IFE, immunofixation electrophoresis; PCs, plasma cells; MDS, myelodysplastic syndrome; MPN, myeloproliferative neoplasm; NGS, next‑generation sequencing; HHV-8, human herpesvirus 8; MCD, multicentric Castleman disease; qPCR, quantitative polymerase chain reaction; NAAT, nucleic acid amplification test; mNGS, metagenomic next-generation sequencing; CMV, cytomegalovirus; SLE, systemic lupus erythematosus; CTD, connective tissue disease; ANA, antinuclear antibody; ENA, extractable nuclear antigen; anti‑dsDNA, anti-double-stranded DNA; ANCA, anti-neutrophil cytoplasmic antibody; MPO, myeloperoxidase; PR3, proteinase 3.
