# Supplementary material for: Case Report: Endothelial-targeted bridging therapy for a TTP-like phenotype in fulminant iMCD-TAFRO
Source: Front Immunol. 2026 Feb 26;17:1776382. doi: 10.3389/fimmu.2026.1776382 (PMC12979455; doi:10.3389/fimmu.2026.1776382)
Supplement: Supplementary Figure 1 — Diagnostic and initial management algorithm for hyperinflammatory TMA/TTP-like presentations with parallel evaluation for iTTP and iMCD/iMCD-TAFRO. Stepwise workflow for patients presenting with TMA/TTP-like features and hyperinflammation. Management is divided into pre-ADAMTS13 (while results are pending) and post-ADAMTS13 (after results return) phases. The pathway integrates early iTTP-directed therapy when clinical suspicion is high, parallel exclusion of secondary TMA causes, and expedited evaluation for iMCD/iMCD-TAFRO (including lymph-node biopsy when feasible) to guide subsequent iTTP- or iMCD-directed treatment escalation. Abbreviations: as defined in the main text and Figure legends. [file SupplementaryFile1.zip › Table S2.docx]

**Supplementary Table S2. Cross-walk to 2021 validated international definition of iMCD-TAFRO**

| **2021 iMCD-TAFRO definition element** | **Requirement** | **Patient-specific evidence (this case)** | **Met?** |
| --- | --- | --- | --- |
| **Pathological criterion (mandatory for definite iMCD‑TAFRO)** | LN consistent with iMCD histopathologic features per international iMCD criteria | LN core biopsy consistent with iMCD spectrum; HHV-8 negative by plasma PCR; EBER negative; polytypic κ/λ | Yes |
| **Clinical criterion T (mandatory)** | Thrombocytopenia: pre-treatment platelet nadir ≤100×10^9^/L | PLT nadir 6×10^9^/L (≤100×10^9^/L) | Yes |
| **Clinical criterion A (mandatory)** | Anasarca: pleural effusion, ascites, or subcutaneous edema | Generalized edema/anasarca; pleural effusions and ascites on imaging; marked abdominal distension | Yes |
| **Clinical criterion F (mandatory)** | Fever ≥37.5°C of unknown etiology or CRP ≥2.0 mg/dL | Fever up to 39°C; hsCRP 180.4 mg/L (meets CRP threshold) | Yes |
| **Clinical criterion O (mandatory)** | Organomegaly: small‑volume lymphadenopathy in two or more regions and/or hepatomegaly and/or splenomegaly (on CT) | Multicentric lymphadenopathy involving ≥2 nodal regions (see Table S1a for stations and short-axis diameters); hepatomegaly and splenomegaly on PET/CT | Yes |
| **Additional “R or BM” requirement (at least one required)** | Renal insufficiency or TAFRO-consistent bone marrow (reticulin fibrosis or megakaryocytic hyperplasia) without alternative diagnosis | Bone marrow biopsy: reticulin fibrosis MF-3 and megakaryocytic findings consistent with reactive change; renal dysfunction not disproportionate | Yes |
| **Exclusion criteria (mandatory)** | Must exclude infectious, autoimmune/rheumatologic, and malignant conditions listed in definition | Exclusion work-up summarized in Table S3 | Yes |

*Adapted from Nishimura et al., Am J Hematol 2021[2].*
