# Supplementary material for: Case Report: Endothelial-targeted bridging therapy for a TTP-like phenotype in fulminant iMCD-TAFRO
Source: Front Immunol. 2026 Feb 26;17:1776382. doi: 10.3389/fimmu.2026.1776382 (PMC12979455; doi:10.3389/fimmu.2026.1776382)
Supplement: Supplementary Figure 1 — Diagnostic and initial management algorithm for hyperinflammatory TMA/TTP-like presentations with parallel evaluation for iTTP and iMCD/iMCD-TAFRO. Stepwise workflow for patients presenting with TMA/TTP-like features and hyperinflammation. Management is divided into pre-ADAMTS13 (while results are pending) and post-ADAMTS13 (after results return) phases. The pathway integrates early iTTP-directed therapy when clinical suspicion is high, parallel exclusion of secondary TMA causes, and expedited evaluation for iMCD/iMCD-TAFRO (including lymph-node biopsy when feasible) to guide subsequent iTTP- or iMCD-directed treatment escalation. Abbreviations: as defined in the main text and Figure legends. [file SupplementaryFile1.zip › Table S1a.docx]

**Supplementary Table S1a. Pre-transfer PET/CT report–derived lymph node stations and short-axis diameters supporting multicentric lymphadenopathy.**

| **Nodal station** | **CT size (cm)** | **Short-axis (cm)** | **SUVmax** |
| --- | --- | --- | --- |
| Left cervical | 1.6 × 0.7 | 0.7 | 5.7 |
| Right cervical | 2.2 × 1.1 | 1.1 | 4.8 |
| Mediastinal | 1.2 × 1.1 | 1.1 | 2.3 |
| Hilar (bilateral) | 1.2 × 0.9 | 0.9 | 1.9 |
| Left axillary | 1.1 × 0.8 | 0.8 | 3.5 |
| Right axillary | 0.9 × 0.8 | 0.8 | 3.6 |
| Abdominal cavity node | 0.6 × 0.6 | 0.6 | 1.1 |
| Para‑aortic | 1.2 × 1.0 | 1.0 | 6.1 |
| External iliac | 1.2 × 0.9 | 0.9 | 2.6 |
| Left inguinal | 0.9 × 0.8 | 0.8 | 3.2 |
| Right inguinal | 1.0 × 0.6 | 0.6 | 2.5 |

**Footnote:** Short-axis was defined as the smaller diameter on CT. Per the 2017 international iMCD diagnostic criteria, multicentric lymphadenopathy requires enlarged lymph nodes (≥1.0 cm short-axis) in ≥2 nodal stations; this requirement was met (e.g., right cervical and mediastinal/para‑aortic nodes).
