# Supplementary material for: Case Report: Endothelial-targeted bridging therapy for a TTP-like phenotype in fulminant iMCD-TAFRO
Source: Front Immunol. 2026 Feb 26;17:1776382. doi: 10.3389/fimmu.2026.1776382 (PMC12979455; doi:10.3389/fimmu.2026.1776382)
Supplement: Supplementary Figure 1 — Diagnostic and initial management algorithm for hyperinflammatory TMA/TTP-like presentations with parallel evaluation for iTTP and iMCD/iMCD-TAFRO. Stepwise workflow for patients presenting with TMA/TTP-like features and hyperinflammation. Management is divided into pre-ADAMTS13 (while results are pending) and post-ADAMTS13 (after results return) phases. The pathway integrates early iTTP-directed therapy when clinical suspicion is high, parallel exclusion of secondary TMA causes, and expedited evaluation for iMCD/iMCD-TAFRO (including lymph-node biopsy when feasible) to guide subsequent iTTP- or iMCD-directed treatment escalation. Abbreviations: as defined in the main text and Figure legends. [file SupplementaryFile1.zip › Table S1.docx]

**Supplementary Table S1. Cross-walk to 2017 International iMCD diagnostic criteria**

| **2017 iMCD criterion** | **Requirement** | **Patient-specific evidence (this case)** | **Met?** |
| --- | --- | --- | --- |
| **Major criterion (1)** | Lymph node (LN) histopathology consistent with the iMCD spectrum | Left inguinal LN core needle biopsy showed iMCD-consistent features: regressed germinal centers; prominent vascular proliferation/dilated sinuses; interfollicular plasmacytosis with CD38/CD138+ plasma cells; polyclonal κ and λ without light-chain restriction; preserved follicular dendritic cell network (CD21+); EBER in situ hybridization negative | Yes |
| **Major criterion (2)** | Multicentric lymphadenopathy: enlarged LNs (≥1 cm short-axis) in ≥2 LN stations | PET/CT showed multicentric lymphadenopathy with ≥2 stations meeting short-axis ≥1.0 cm (right cervical 1.1 cm; mediastinal 1.1 cm; para‑aortic 1.0 cm), see Table S1a. | Yes |
| **Minor criteria – Clinical** | Clinical minor criteria include: (1) constitutional symptoms (night sweats, fever >38°C, weight loss, or fatigue), (2) hepatosplenomegaly, (3) fluid accumulation (edema/anasarca/ascites/pleural effusion), (4) eruptive cherry hemangiomatosis or violaceous papules, (5) lymphocytic interstitial pneumonitis (LIP) | Fatigue; hepatosplenomegaly on imaging; anasarca with pleural/peritoneal/pericardial effusions; pulmonary involvement present on CT (bilateral ground-glass opacities/crazy-paving), not definitively classified as LIP | Yes |
| **Minor criteria – Laboratory** | Laboratory minor criteria include: elevated CRP/ESR, anemia, thrombocytopenia/thrombocytosis, hypoalbuminemia, renal dysfunction/proteinuria, polyclonal hypergammaglobulinemia | Elevated CRP/hsCRP (14.54→180.4 mg/L); anemia (HGB nadir ~50 g/L); thrombocytopenia (PLT nadir 6×10^9^/L); hypoalbuminemia (19.26 g/L); mild renal impairment at presentation (Cr 109 μmol/L; eGFR 65 mL/min/1.73 m^2^); Polyclonal hypergammaglobulinemia present (serum protein electrophoresis: γ-globulin 14.1 g/L [ULN 13.5], γ fraction 30.1% [ULN 18.8]; immunofixation electrophoresis: no monoclonal band). | Yes |
| **Minor criteria rule** | Need ≥2 minor criteria total, with ≥1 laboratory | Met (multiple clinical + multiple laboratory minor criteria) | Yes |
| **Exclusion criteria** | Must exclude malignant, autoimmune/autoinflammatory, and infectious disorders that can mimic iMCD | Exclusion work-up summarized in Table S3 | Yes |

*Adapted from Fajgenbaum et al. Blood. 2017[3].*
