# Supplementary figures and images for: Case Report: Endothelial-targeted bridging therapy for a TTP-like phenotype in fulminant iMCD-TAFRO
Source: Front Immunol. 2026 Feb 26;17:1776382. doi: 10.3389/fimmu.2026.1776382 (PMC12979455; doi:10.3389/fimmu.2026.1776382)

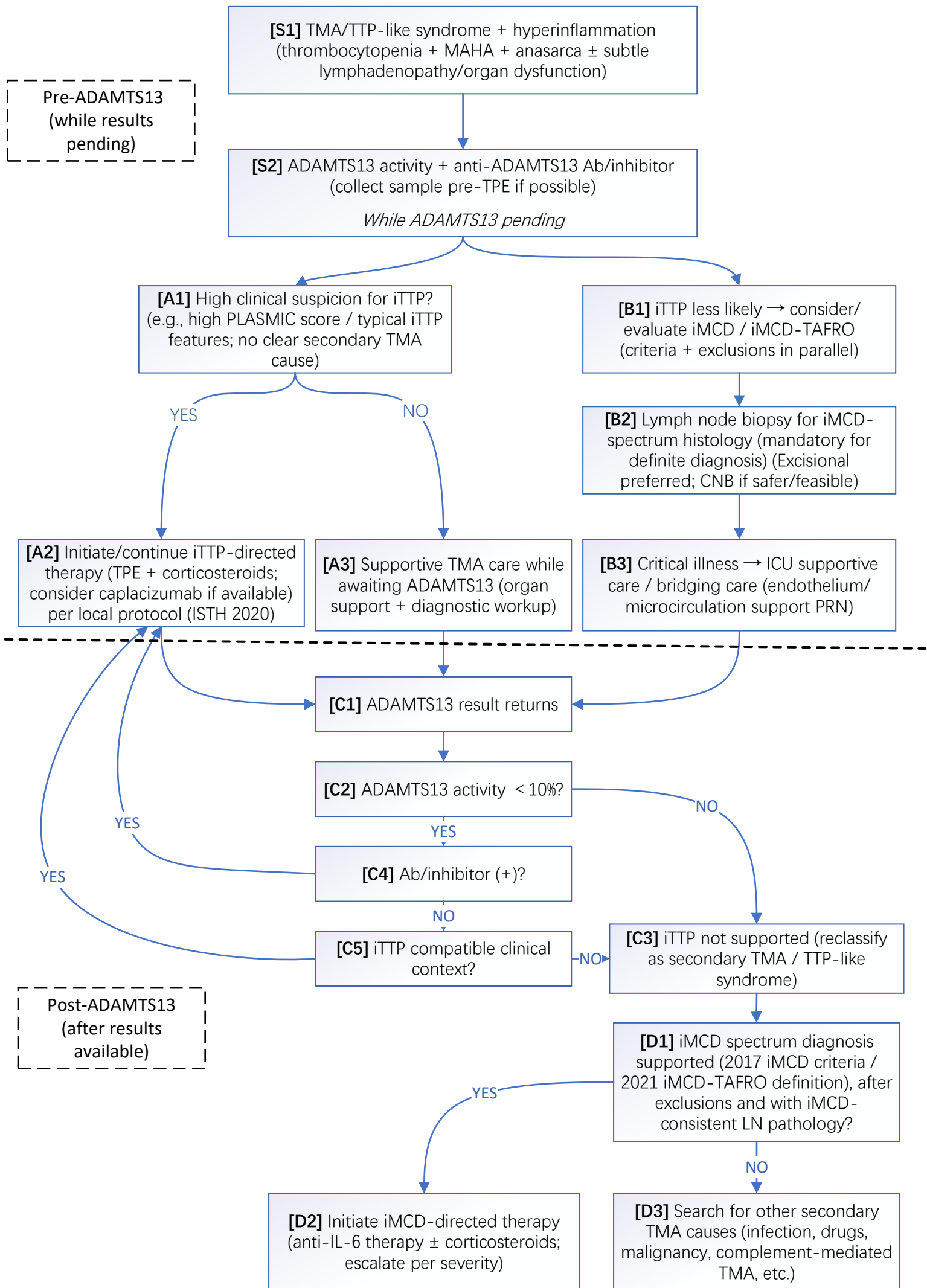

Supplement: Supplementary Figure 1 — Diagnostic and initial management algorithm for hyperinflammatory TMA/TTP-like presentations with parallel evaluation for iTTP and iMCD/iMCD-TAFRO. Stepwise workflow for patients presenting with TMA/TTP-like features and hyperinflammation. Management is divided into pre-ADAMTS13 (while results are pending) and post-ADAMTS13 (after results return) phases. The pathway integrates early iTTP-directed therapy when clinical suspicion is high, parallel exclusion of secondary TMA causes, and expedited evaluation for iMCD/iMCD-TAFRO (including lymph-node biopsy when feasible) to guide subsequent iTTP- or iMCD-directed treatment escalation. Abbreviations: as defined in the main text and Figure legends. [file SupplementaryFile1.zip › Figure S1.PDF]

SC-

27/07/2024 10:55:37  
27/07/2024 14:58:57

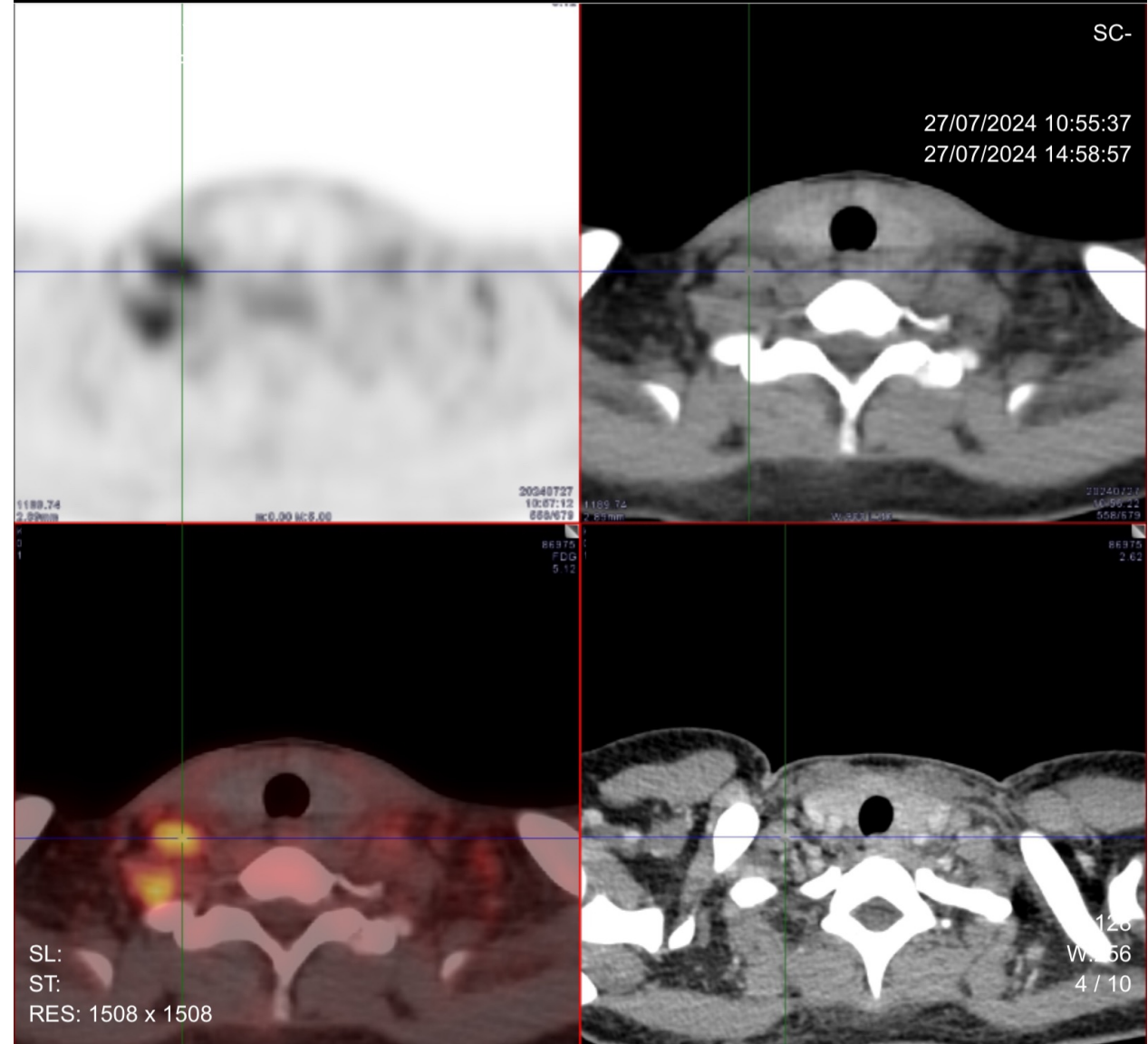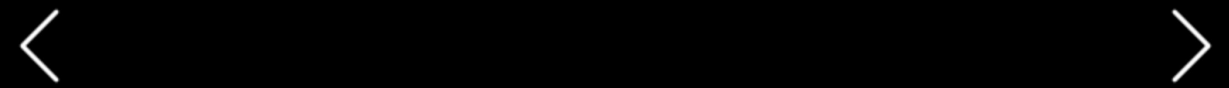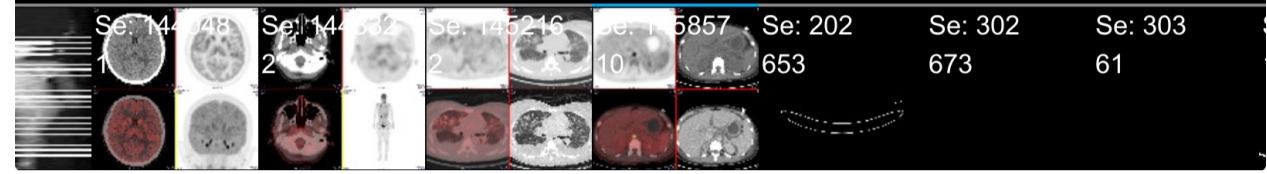

SC-

27/07/2024 10:55:37  
27/07/2024 14:58:57

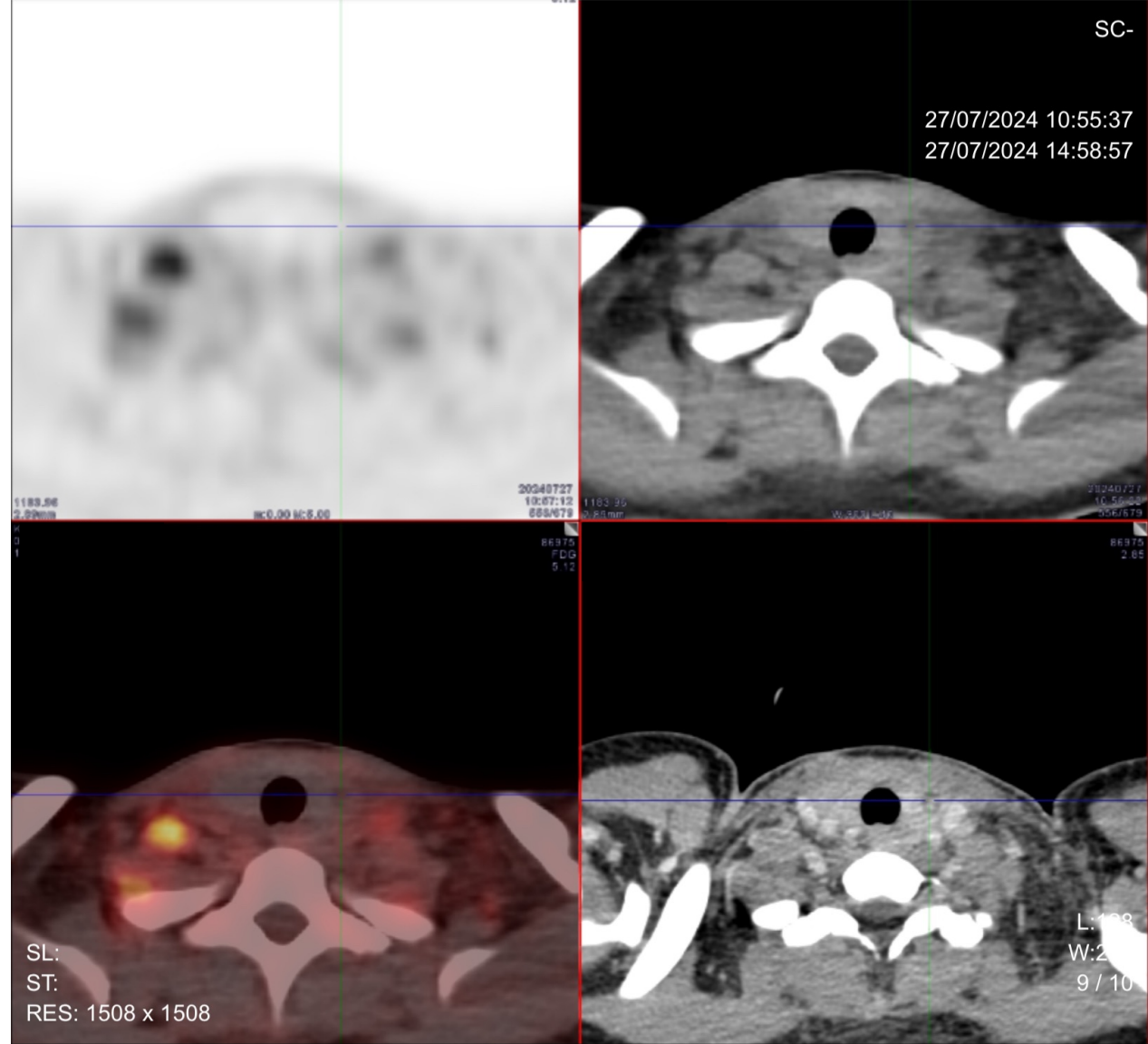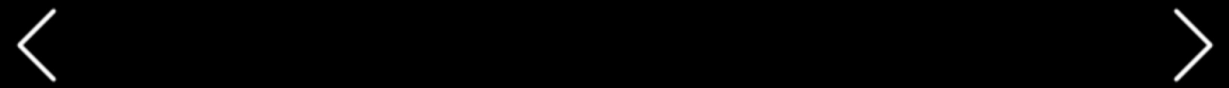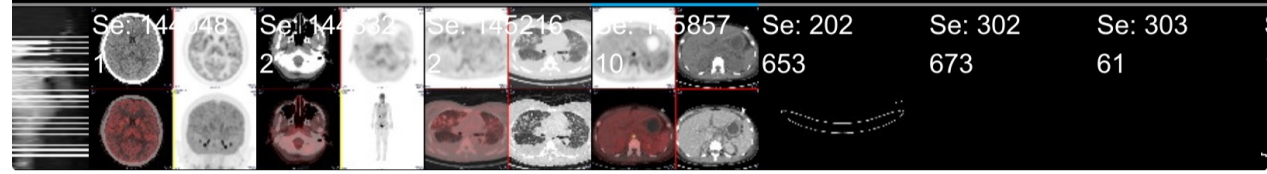

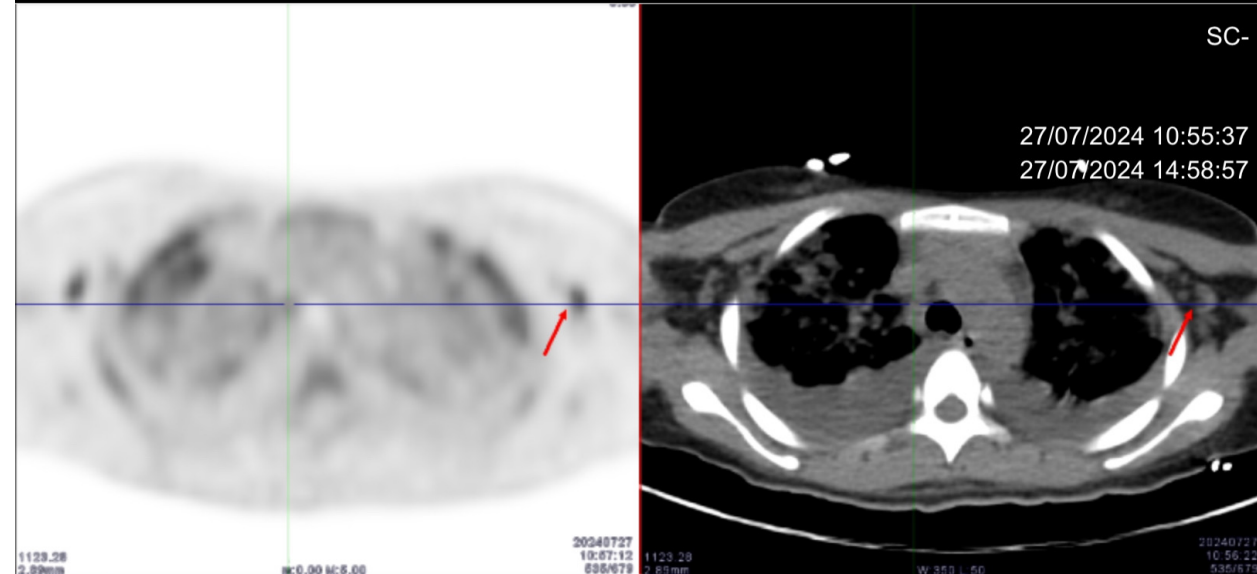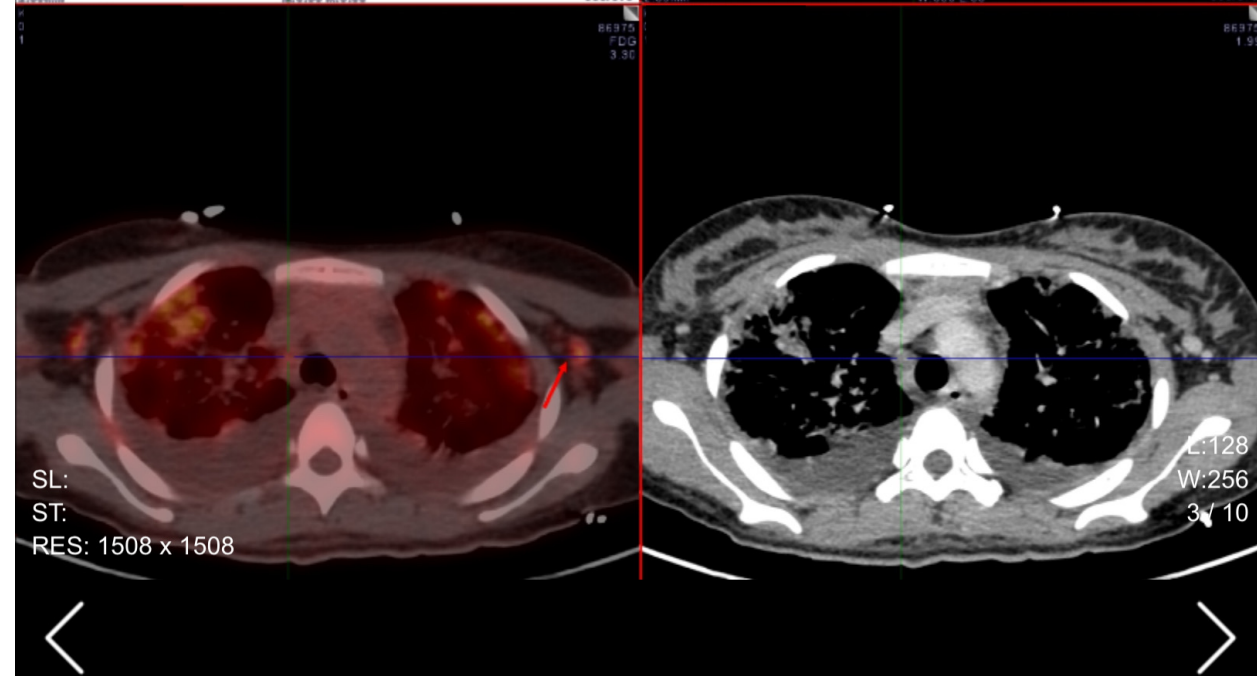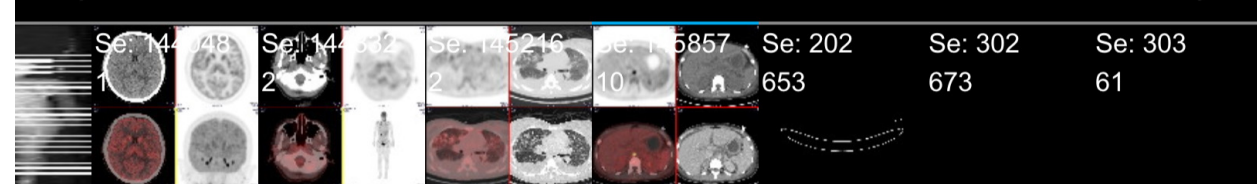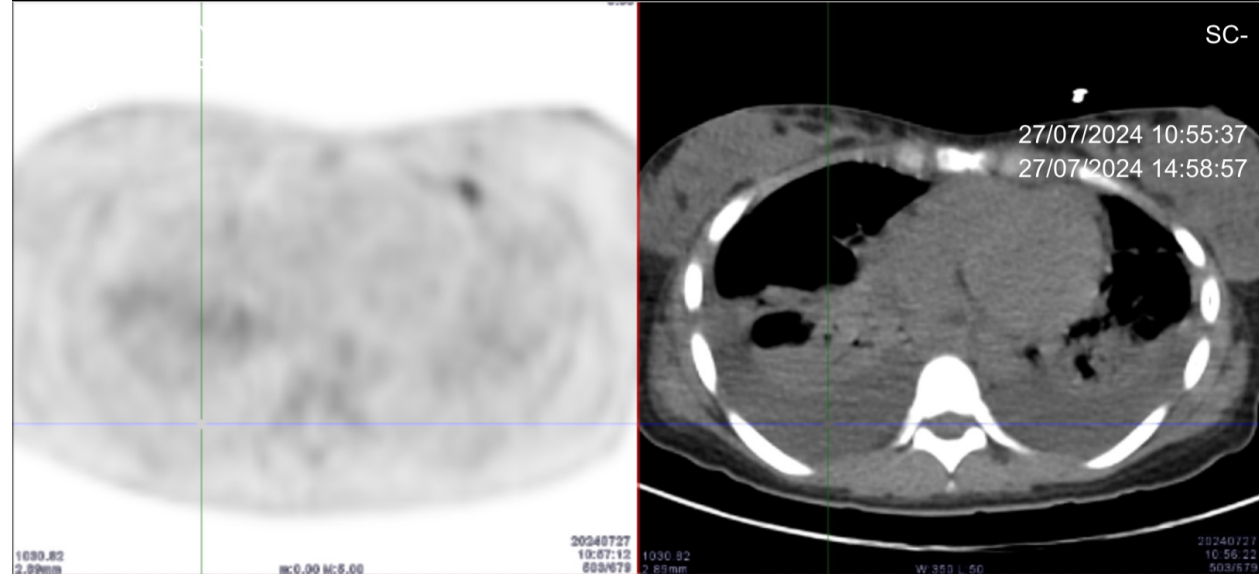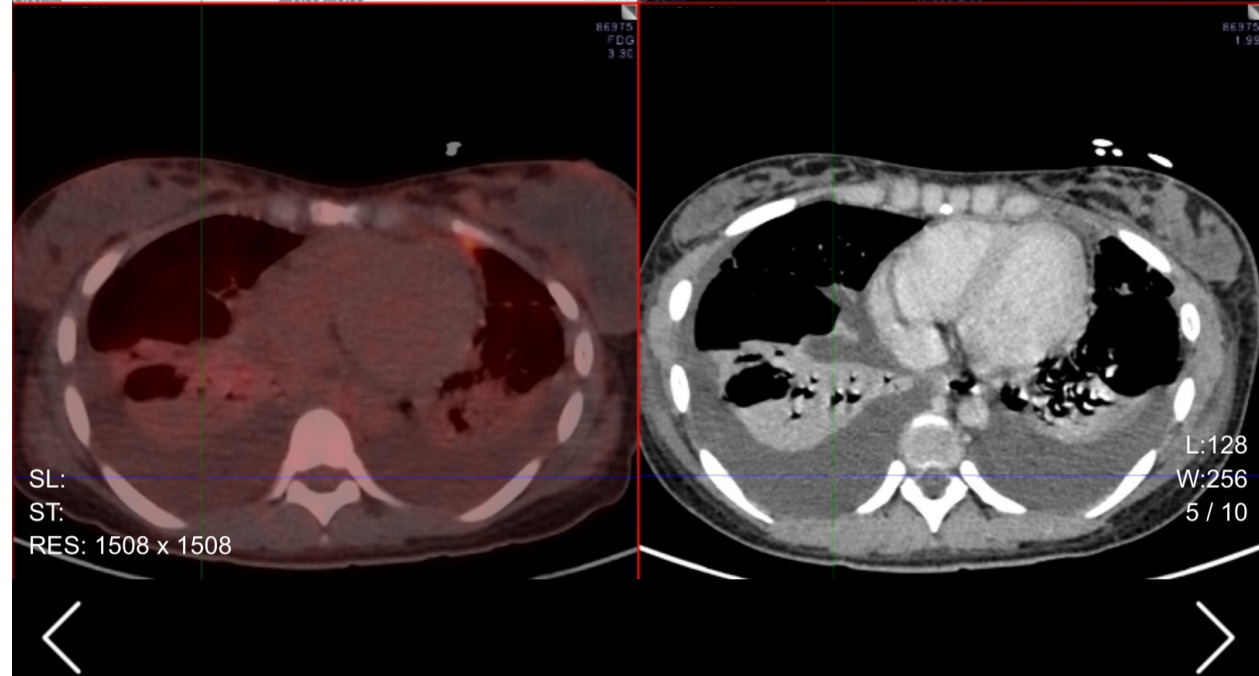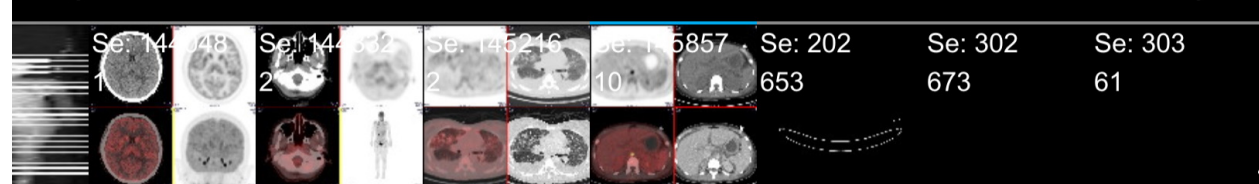

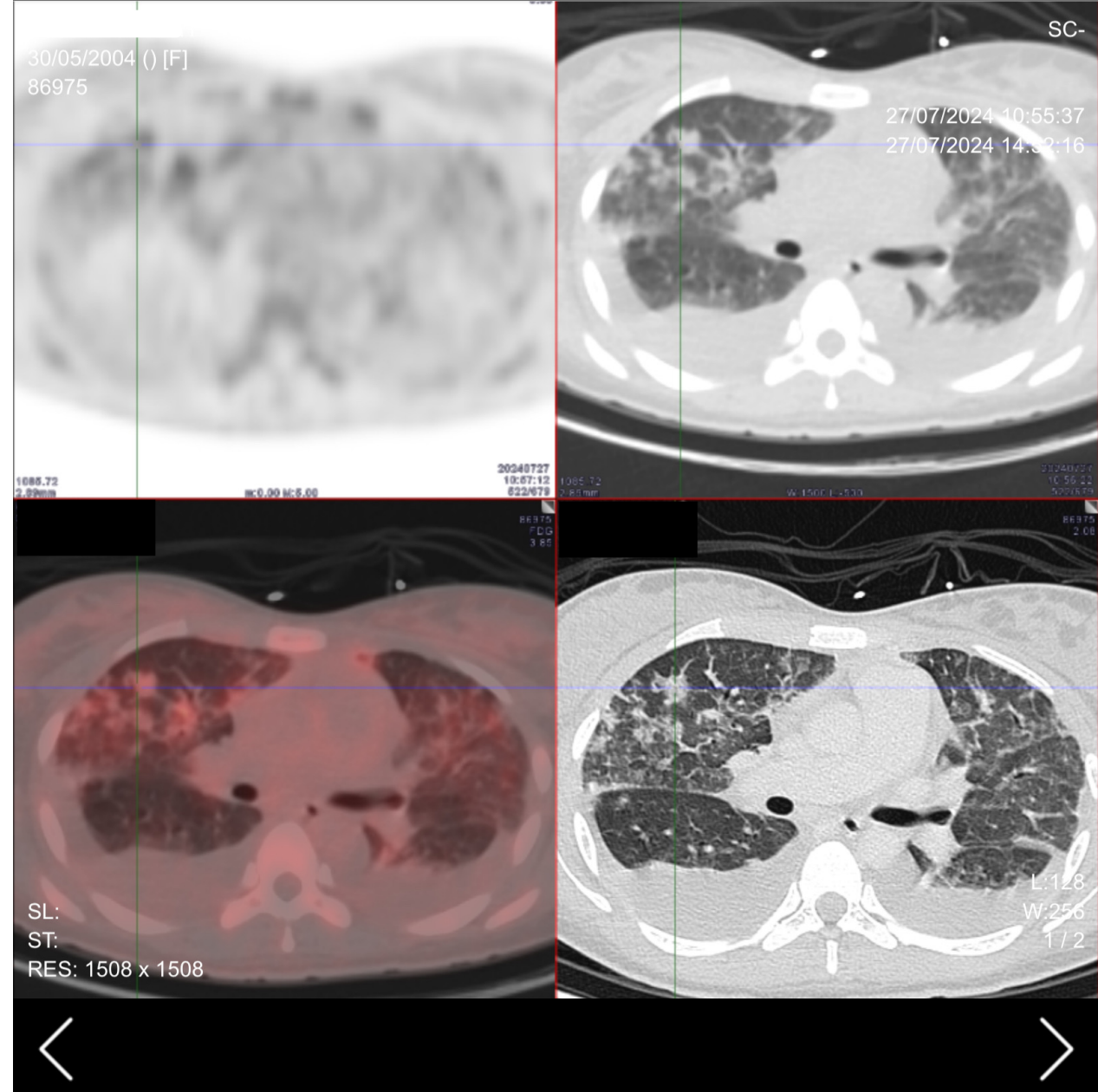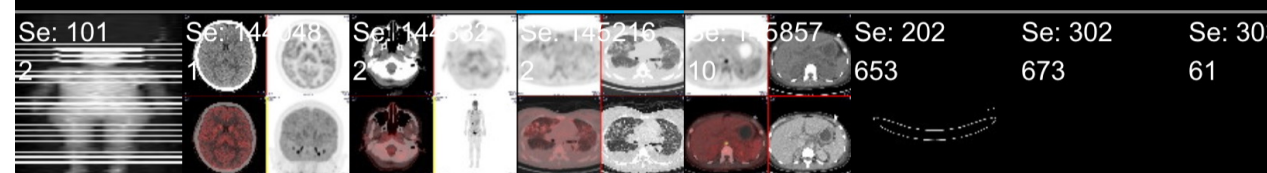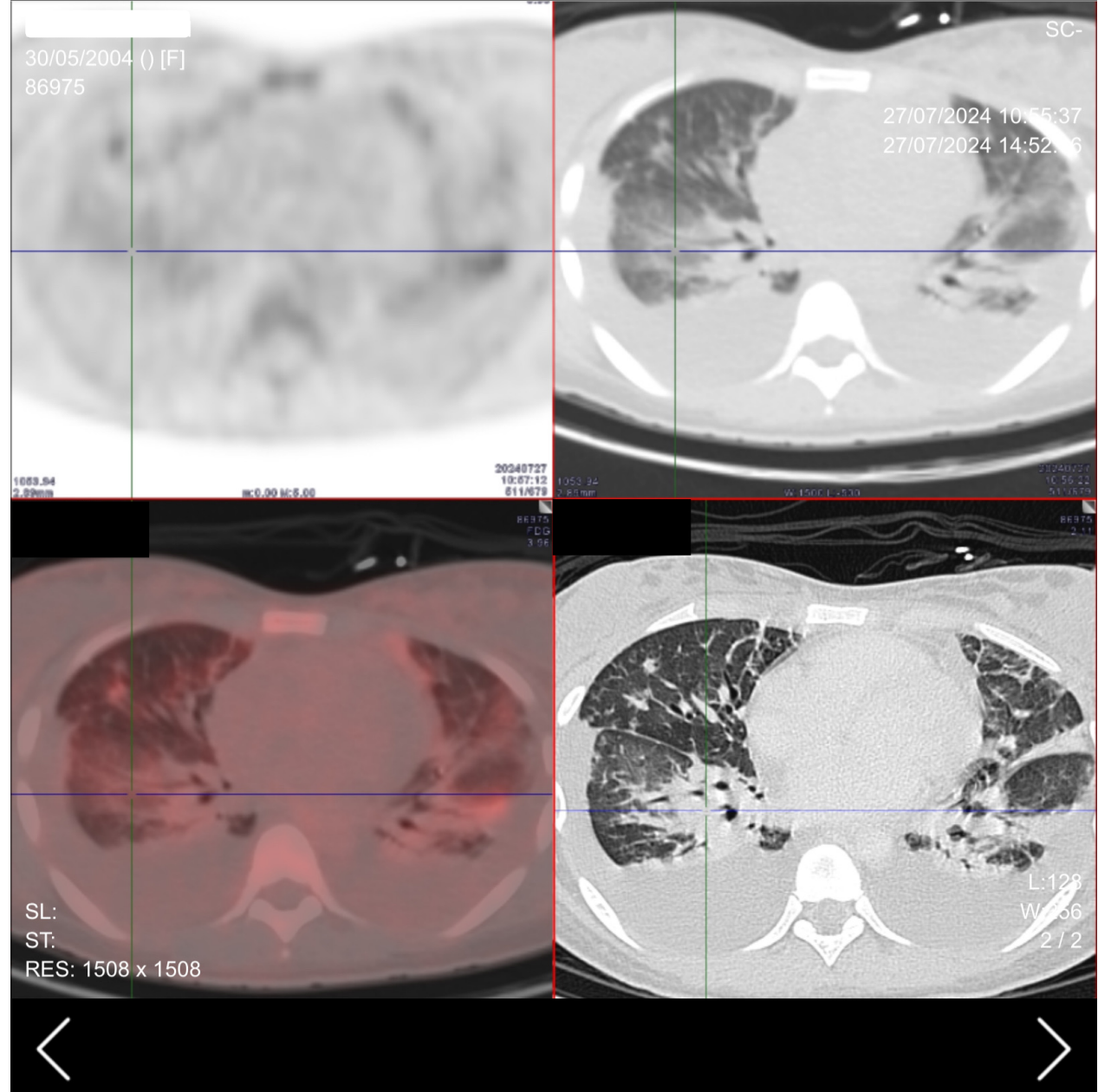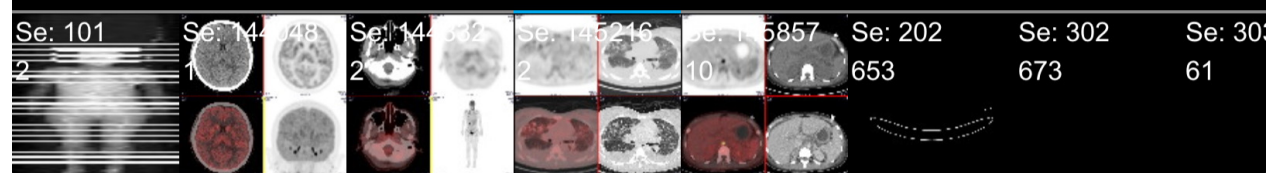

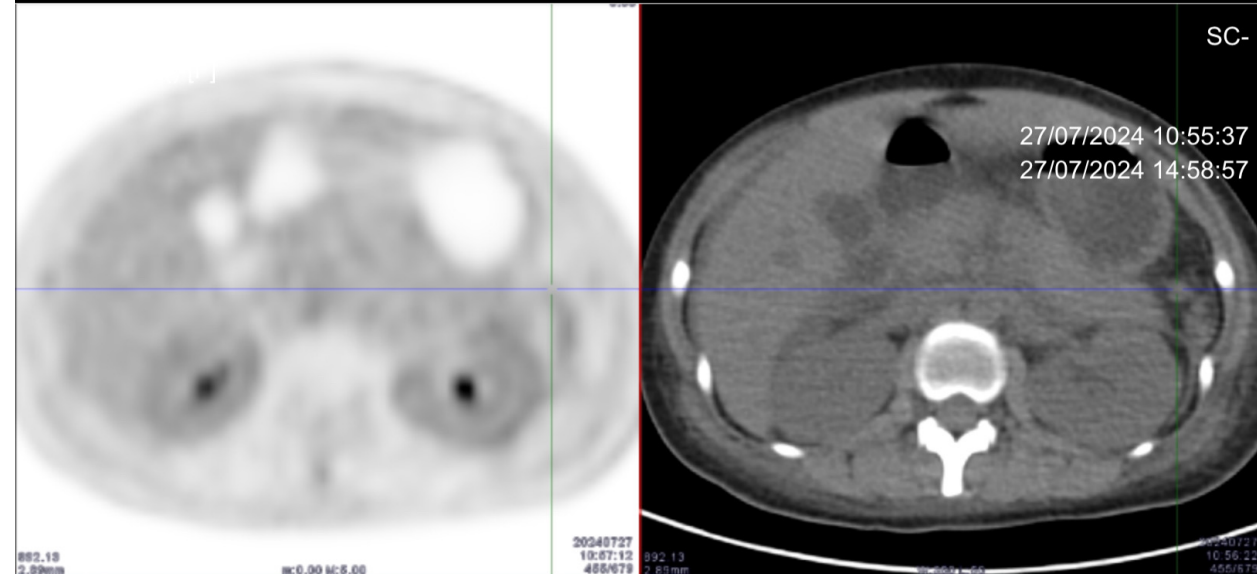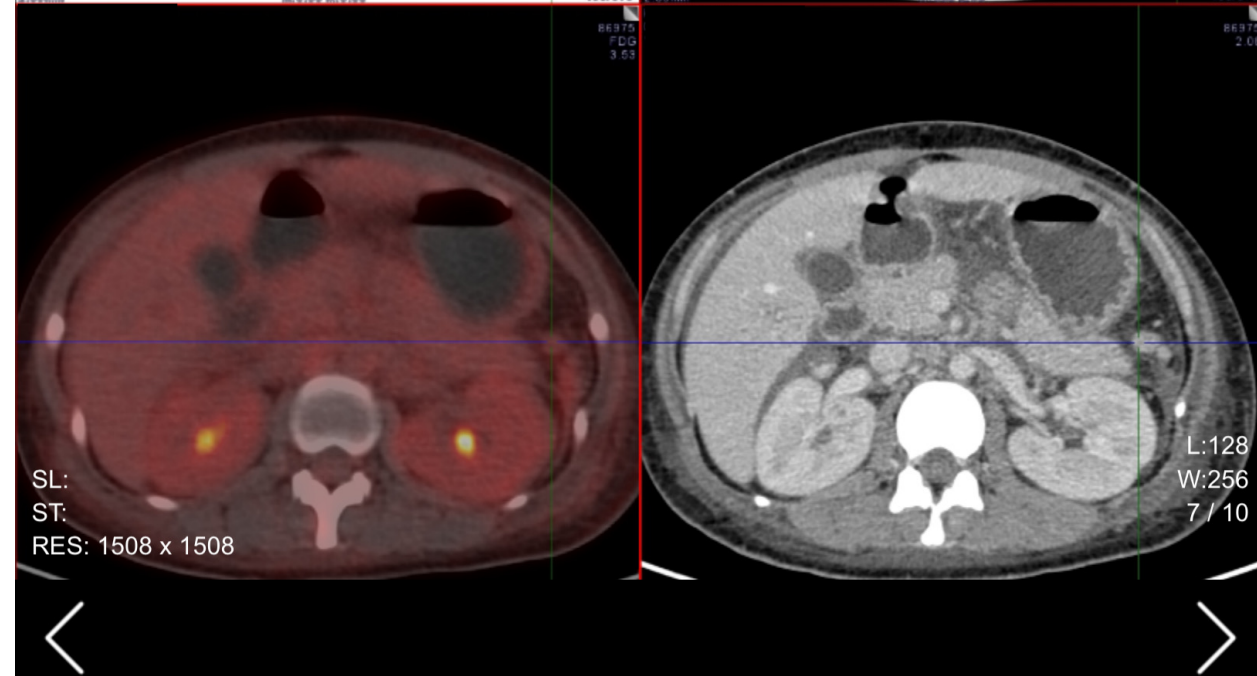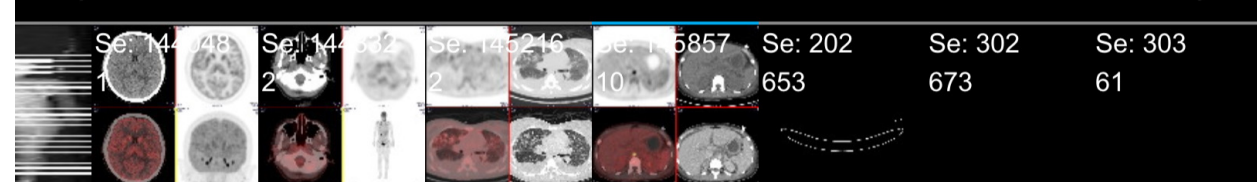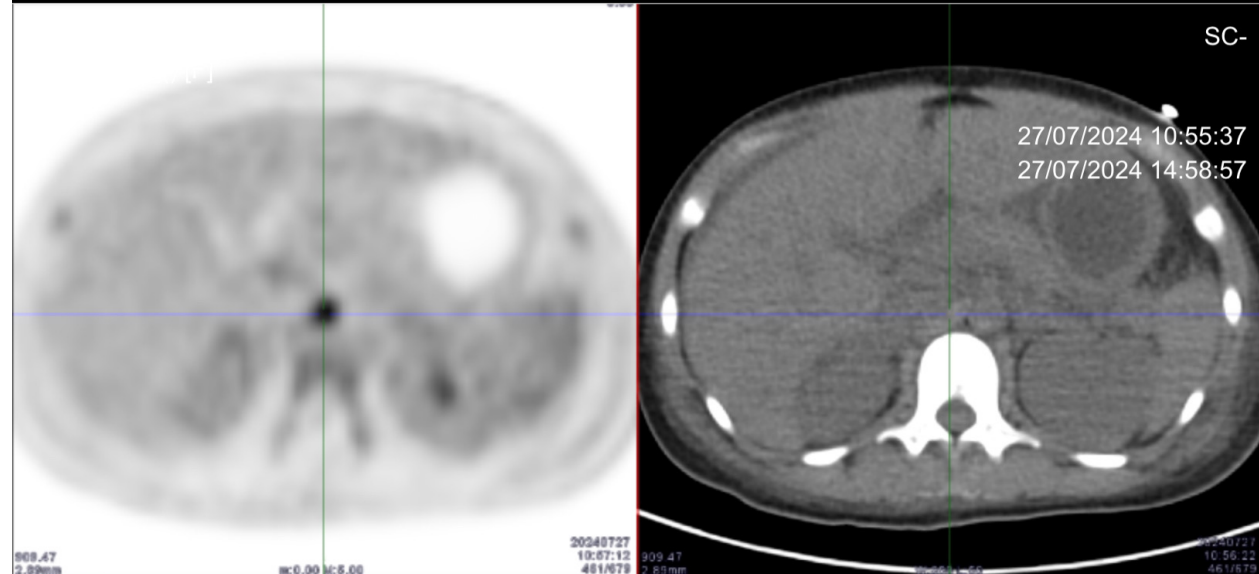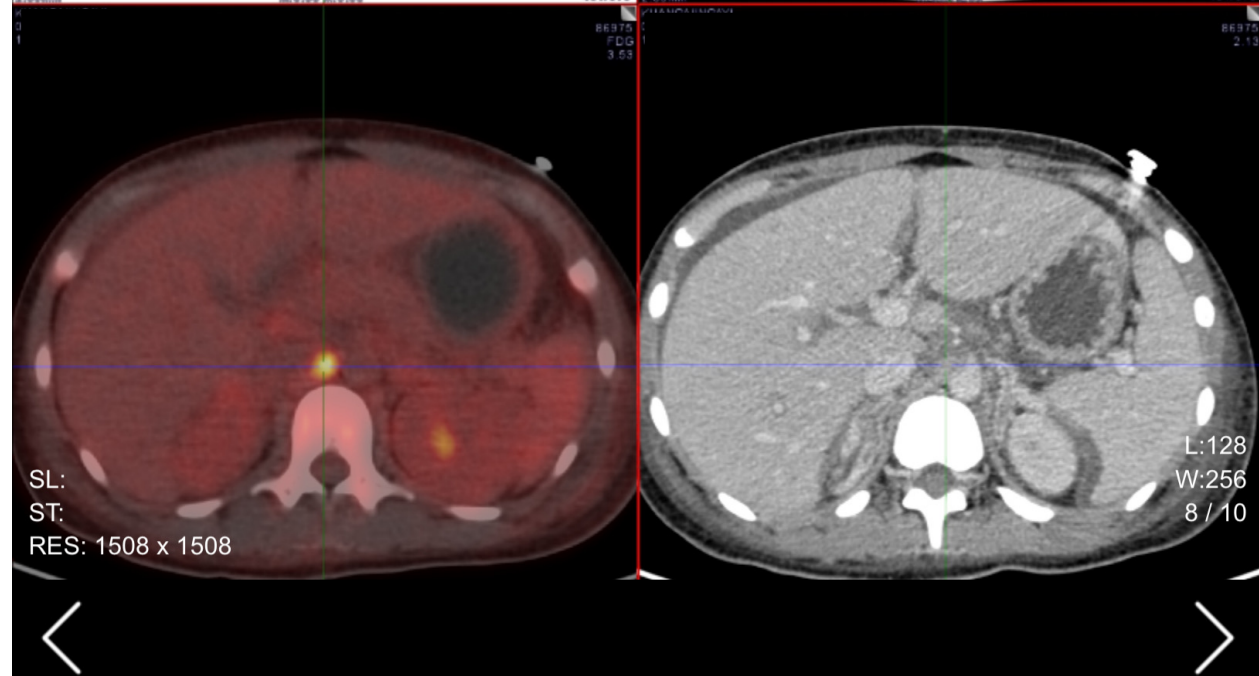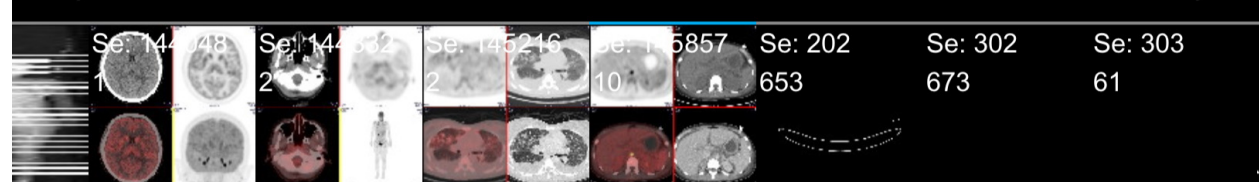

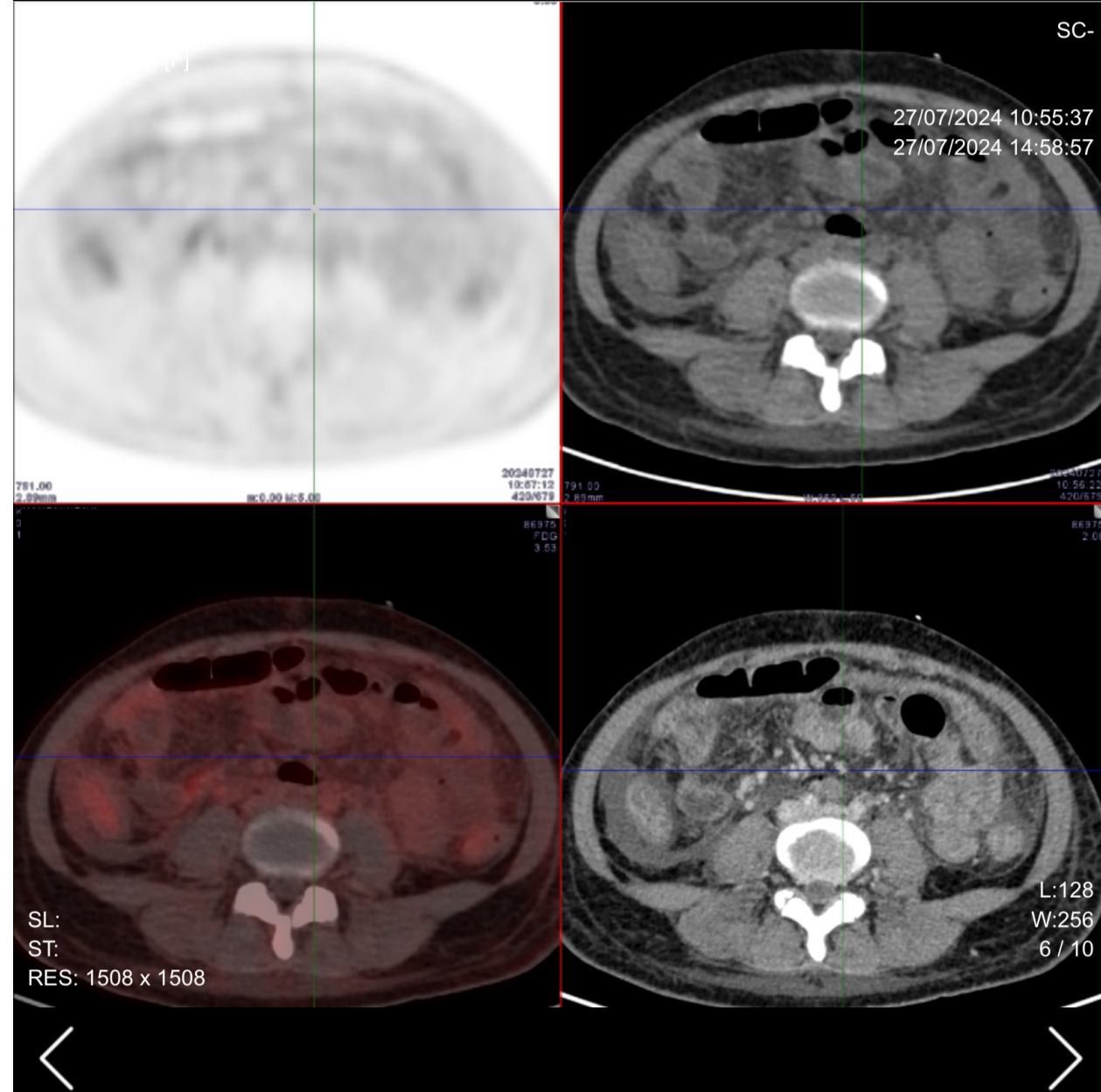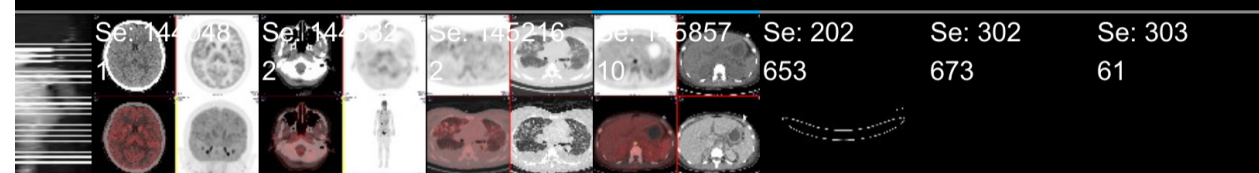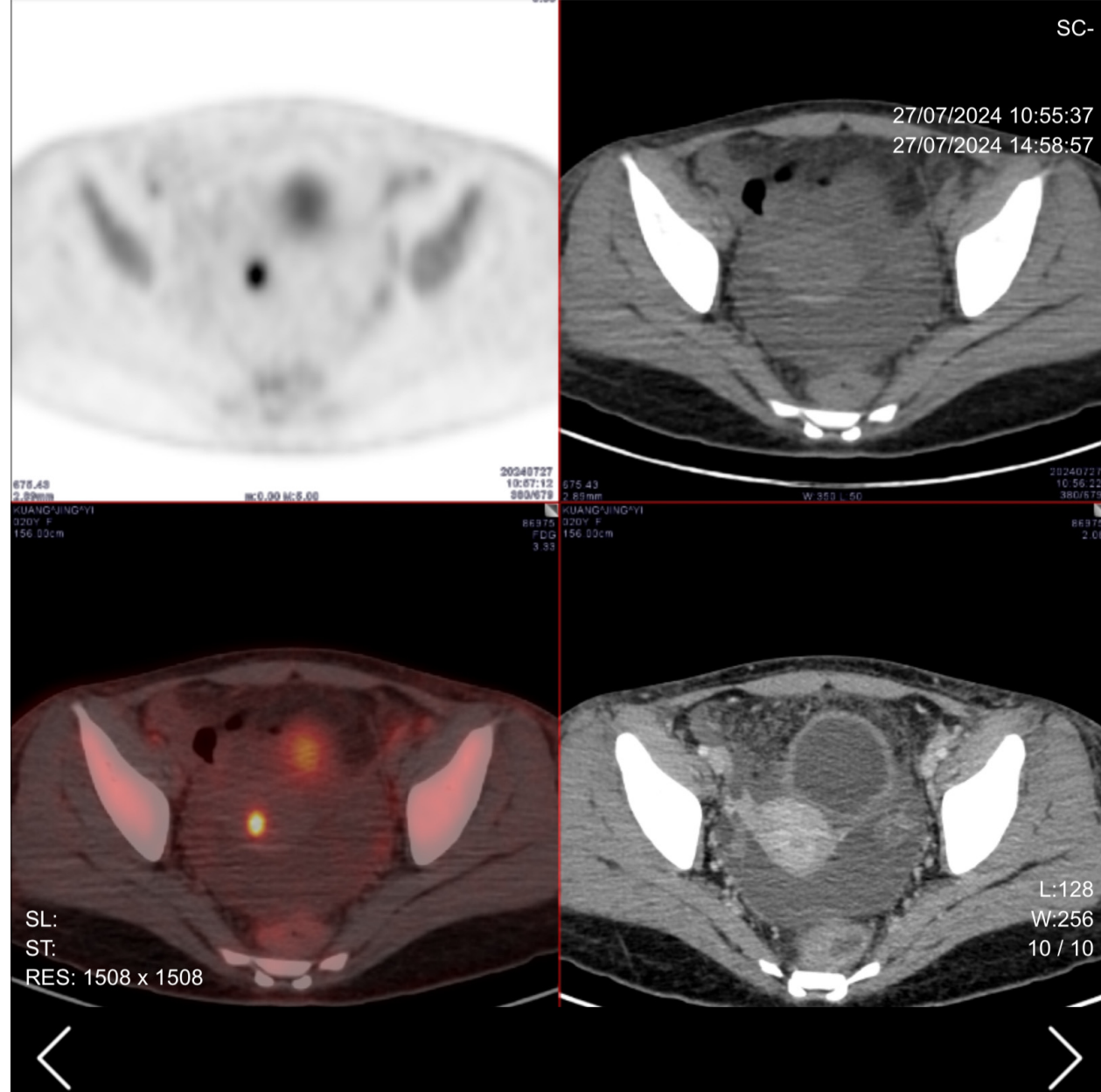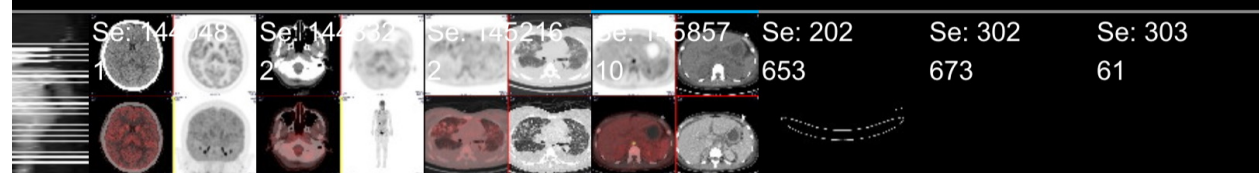

Supplement: Supplementary Figure 1 — Diagnostic and initial management algorithm for hyperinflammatory TMA/TTP-like presentations with parallel evaluation for iTTP and iMCD/iMCD-TAFRO. Stepwise workflow for patients presenting with TMA/TTP-like features and hyperinflammation. Management is divided into pre-ADAMTS13 (while results are pending) and post-ADAMTS13 (after results return) phases. The pathway integrates early iTTP-directed therapy when clinical suspicion is high, parallel exclusion of secondary TMA causes, and expedited evaluation for iMCD/iMCD-TAFRO (including lymph-node biopsy when feasible) to guide subsequent iTTP- or iMCD-directed treatment escalation. Abbreviations: as defined in the main text and Figure legends. [file SupplementaryFile1.zip › Figure S2.pdf]
